# Molecular self-assembly mediates the flocculation activity of benzimidazole derivatives against *E. coli*

**DOI:** 10.1038/s41598-025-13837-z

**Published:** 2025-08-05

**Authors:** Isalyne Drewek, Aurélie Pietka, Thi Quynh Tran, Marharyta Blazhynska, Adéla Jeništova, Christophe Chipot, Andreas Barth, Mathieu Surin, Philippe Leclère, Ruddy Wattiez, Robert N. Muller, Dimitri Stanicki, Sophie Laurent

**Affiliations:** 1 General, Organic and Biomedical Chemistry Unit (CGOB), Laboratory of Nuclear Magnetic Resonance and Molecular Imaging, Faculty of Medicine and Pharmacy, University of Mons - UMONS, Mons, 7000 Belgium; 2 Laboratory of Proteomics and Microbiology, University of Mons - UMONS, Mons, 7000 Belgium; 3 Laboratory for Physics of Nanomaterials and Energy, Research Institute for Materials, University of Mons - UMONS, Mons, 7000 Belgium; 4 Laboratoire International Associé Centre National de la Recherche Scientifique, University of Illinois at Urbana-Champaign, Unité Mixte de Recherche No 7019, Université de Lorraine, F-70239, Vandoeuvre-lès-Nancy Cedex, 54506 France; 5 Department of Biochemistry and Biophysics, The Arrhenius Laboratories for Natural Sciences, Stockholm University, Stockholm, 106 91 Sweden; 6 Department of Biochemistry and Molecular Biology, The University of Chicago, Chicago, IL 60637 USA; 7 Theoretical and Computational Biophysics Group, Beckman Institute, Department of Physics, University of Illinois at Urbana-Champaign, Urbana, IL 61801 USA; 8 Laboratory for Chemistry of Novel Materials, Centre of Innovation and Research in Materials and Polymers (CIRMAP), University of Mons - UMONS, Mons, 7000 Belgium; 9 Center for Microscopy and Molecular Imaging (CMMI), Charleroi, 6041 Belgium

**Keywords:** Benzimidazole derivatives, Bacterial flocculation, *E. coli*, Molecular self-assembly, Biotechnology, Chemical biology, Microbiology, Chemistry, Nanoscience and technology

## Abstract

Bacterial flocculation is a process in which bacteria aggregate to form cloudy, flake-like clusters known as flocs. While this phenomenon is commonly associated with water treatment, it also has interesting industrial applications, particularly as a method for cell immobilisation. *Escherichia coli*, extensively employed in industrial processes, typically does not possess inherent flocculation ability. In this study, we found that certain bisbenzimidazole derivatives can rapidly induce flocculation in *E. coli* (K-12 MG1655) in a structure-dependent manner. Among others, high-resolution microscopy (SEM, fluid AFM) revealed a dense fibrillar network within the flocs, initially suggestive of an extracellular matrix. Mechanistic investigations demonstrated that this phenomenon cannot be linked to the secretion of extracellular polymeric substances (EPS). Our findings suggest that flocculation arises from the self-assembly of bisbenzimidazole derivatives into supramolecular fibres that anchor to bacterial membranes. These results uncover an atypical flocculation process distinct from charge neutralisation or EPS-mediated pathways, broadening the potential applications of bisbenzimidazole derivatives in bacterial immobilisation.

**Supplementary Information:**

The online version contains supplementary material available at 10.1038/s41598-025-13837-z.

## Introduction

Bacteria play a key role in a wide range of industrial applications, including food production^1^, wastewater treatment^2^, biopolymer synthesis^3^, and the production of therapeutic molecules such as hormones^4,5^, antibacterial^6^, or anti-cancer agents^7^. The industrial use of bacterial systems has expanded significantly in recent years, driven by technological advances and the growing demand for sustainable and efficient bioprocesses. In this context, research is focusing on optimising these processes to improve their efficiency. One promising approach is cell immobilisation *via* flocculation, which offers several advantages over the use of planktonic (free-floating) cells^8–10^.

A widely recognised application of bacterial flocculation is in wastewater treatment, where activated sludge relies heavily on microbial flocs. These flocs are composed of bacteria embedded in a matrix of extracellular polymeric substances (EPS), including polysaccharides, proteins, and nucleic acids. They play a key role in pollutant removal and organic matter degradation under both aerobic and anaerobic conditions^11,12^. Beyond wastewater treatment, flocculation has demonstrated significant potential in various industrial bioprocesses. Early studies showed that flocculation-based immobilisation of *Zymomonas mobilis* enabled continuous ethanol production with enhanced productivity. In glucose-rich media, Ghommodh and Bulock reported an eightfold increase in ethanol output compared to planktonic cultures^13,14^. More recently, Ojima et al. investigated ethanol production in flocculated *E. coli* strain KO11, showing high yields through fermentation with easy cell recovery after floc sedimentation, and allowing use in repeated batch cultures^15^. Bacterial flocculation has also shown promise in chemical synthesis. For example, Rehn et al. studied flocculated *E. coli* cells containing ω-transaminase for the asymmetric synthesis of (S)-4’-cyano-α-methylbenzylamine. The process demonstrated high efficiency in target molecule production, maintaining high catalytic performance across five successive reaction cycles^16^.

In the literature, bacterial flocculation is generally described as an aggregation process in which microbial cells cluster together to form aggregates with a cloudy, flaky appearance. While some bacterial strains naturally exhibit flocculation abilities^17,18^, others require induced flocculation. This is the case for *E. coli*, a bacterium widely used in industry, which lacks inherent flocculating properties. Induced flocculation can be achieved through three main strategies: (**I**) adding flocculating agents such as inorganic compounds, synthetic polymers, or bio-based polymers; (**II**) modifying the bacterial phenotype *via* physical treatment or genetic engineering; (**III**) co-culturing with a naturally flocculating microorganism^19^. From a mechanistic point of view, flocculation is generally attributed to either bridging, where EPS or other macromolecules form a network linking bacterial cells, or charge neutralisation, where positively charged flocculants (e.g., polyethyleneimine^20–22^ or chitosan^16,23–25^ counteract the negative charge of bacterial surfaces^26^. Each approach has its limitations: genetic modification raises regulatory concerns, while polycationic agents may lose effectiveness under varying environmental conditions^19^.

**Fig. 1:**
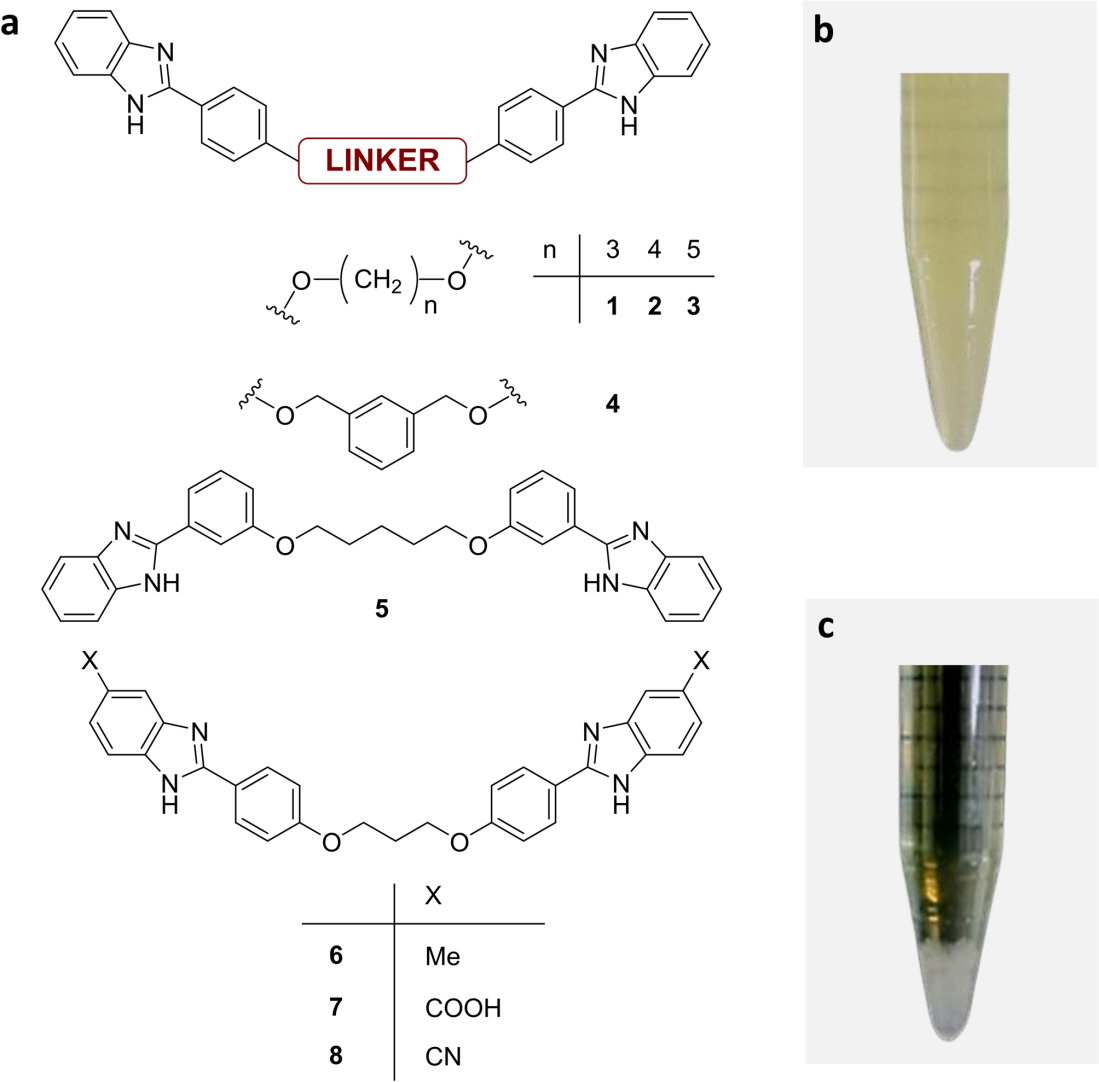
(**a**) Bisbenzimidazole derivatives considered in the SAR study to investigate the observed flocculation phenomenon in the photographs (on the right). (**b**) Observation of an *E. coli* bacterial culture, (**c**) observation of the bacterial pellet deposited at the bottom of the tube after flocculation induction.

In our search for novel antibacterial derivatives, we focused on bisbenzimidazole structures (Fig. 1, a). Although we did not initially expect these structurally simple derivatives to trigger bacterial flocculation (for example, by neutralising surface charges), liquid culture experiments with *E. coli* have unexpectedly revealed their ability to induce rapid (within minutes) and efficient flocculation (Fig. 1, b-c).

Given the increasing interest in bacterial flocculation, not only for wastewater treatment but also for optimising various industrial processes^13,14,16^ (e.g., biocatalysis, biodegradation) through cell immobilisation, we aimed to expand previous studies to deepen our understanding of this unexpected flocculation process. Our initial step involved a structure-activity relationship (SAR) study to identify a compound with optimal properties. The derivative exhibiting the most promising flocculation profile was then selected to elucidate the flocculation mechanism induced by bisbenzimidazole derivatives.

## Results and discussion

### Derivatives synthesis and assessment of flocculation efficiency

**Fig. 2:**
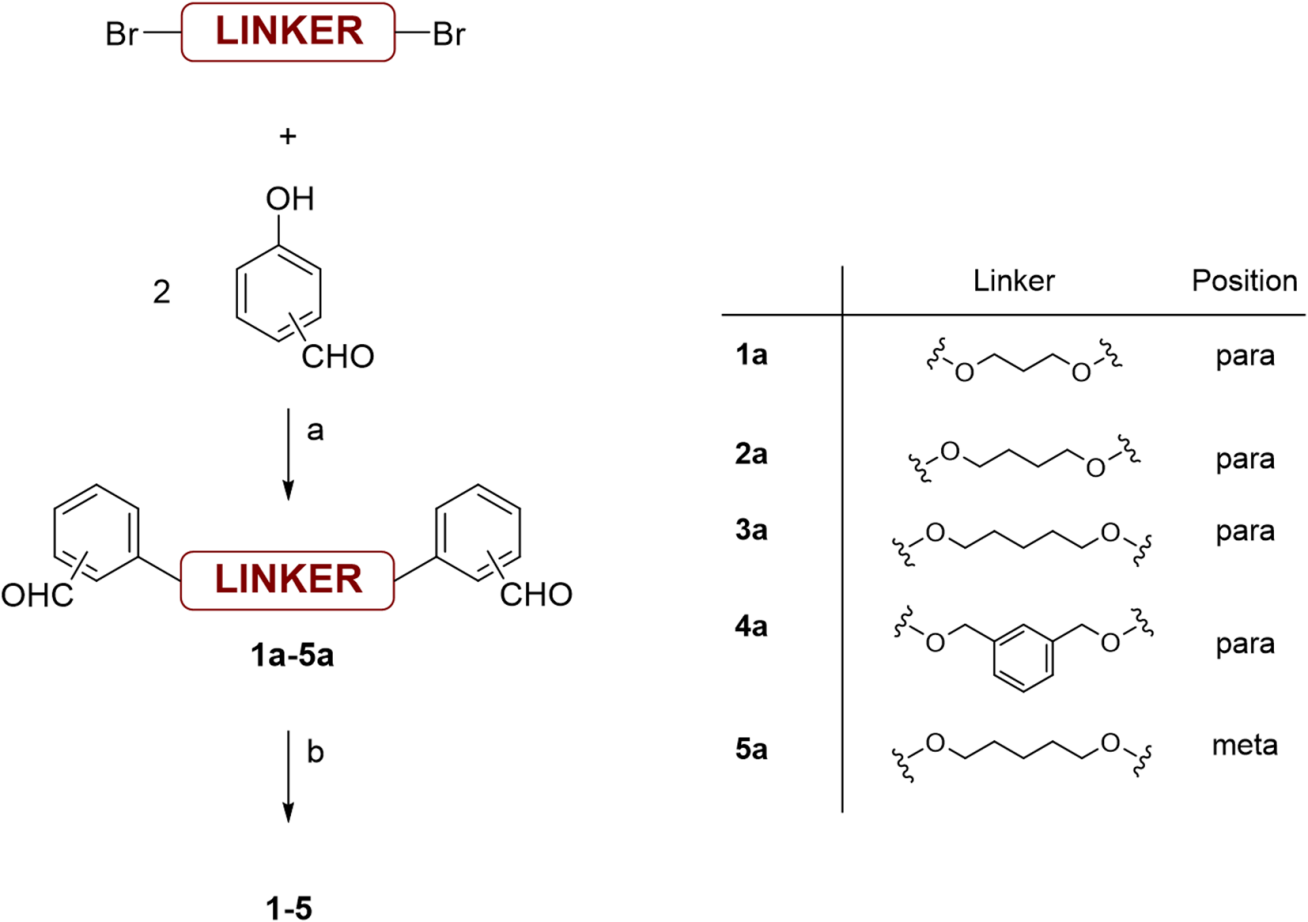
Preparation of compounds **1**–**5**. Reaction conditions: (**a**) EtOH, Na_2_CO_3_ (2 equiv.), reflux 8 h, (**b**) EtOH/H_2_O (1:1), Na_2_S_2_O_5_ (2 equiv.), 1,2-phenylenediamine (2 equiv.), reflux 8 h.

Structures **1**–**5** were synthesised through a two-step procedure^27^ (Fig. 2). First, the corresponding bisbenzaldehyde was prepared by treating the corresponding α,ω-dibromoalkane with two equivalents of 4-hydroxybenzaldehyde in hot ethanol in the presence of Na₂CO₃. After isolation, the resulting dialdehydes were treated with *o*-phenylenediamine in the presence of sodium metabisulfite, according to a procedure described by Mayence et al.^27^. The global yields range from 50 to 98%.

The flocculation activity was evaluated using the K-12 MG1655 strain of *E. coli*, a non-pathogenic, Gram-negative model bacterium commonly used in laboratories. The method involved monitoring and measuring the optical density (O.D.) of bacterial cultures (i.e., supernatant) over time following treatment with the test compound. To ensure consistent results, all experiments were performed with a final standardised compound concentration of 59 µM and a fixed DMSO percentage of 5.9%. These conditions were optimised in advance through experimental evaluations. Under these parameters, DMSO had a minimal impact on bacterial growth (Fig. S1), while most of the compounds remained soluble throughout the experiment.

**Fig. 3:**
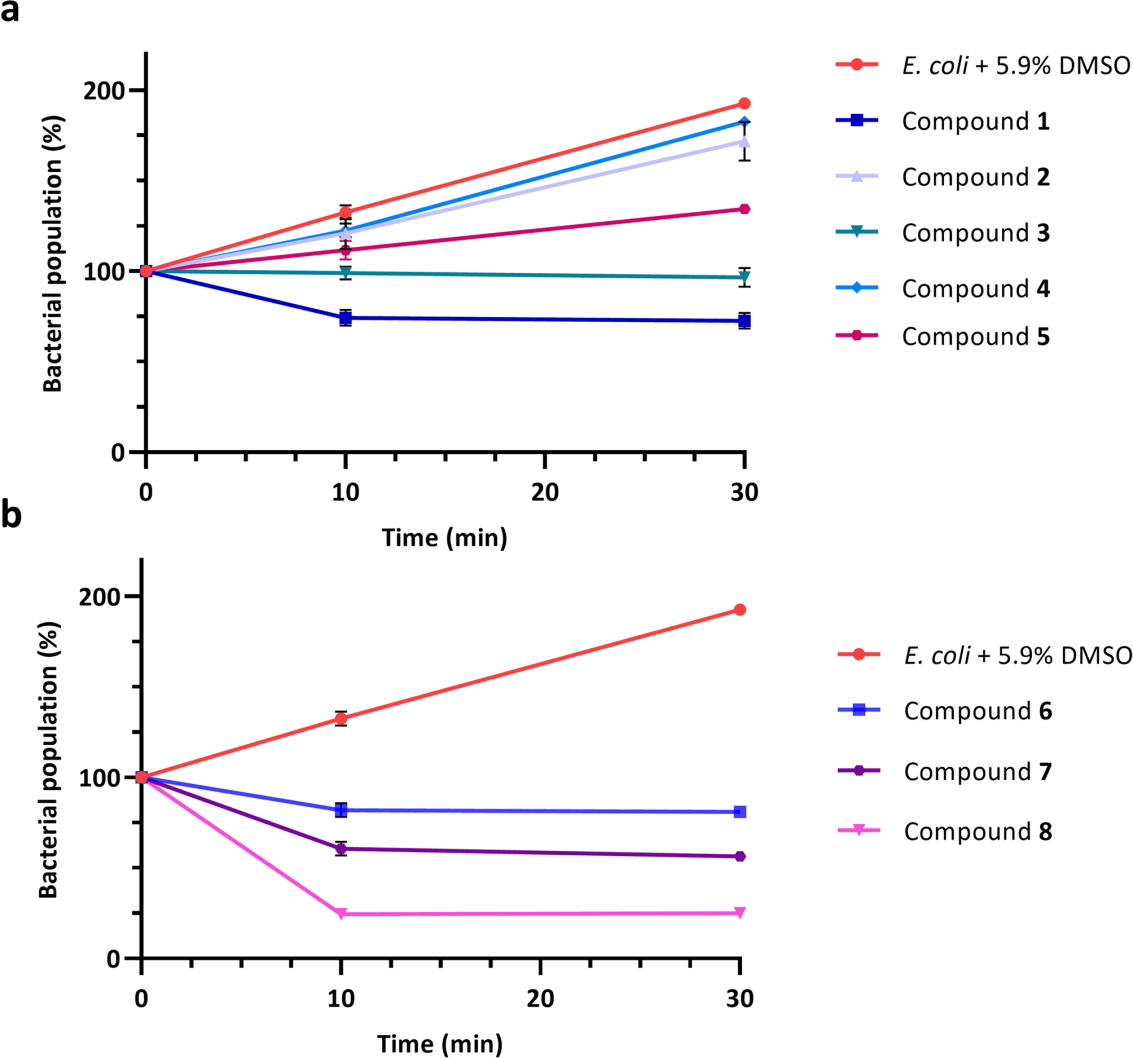
Results of the SAR investigation on *E. coli* (K-12 MG1655). The graphs show normalised optical density (O.D. 600 nm) measurements of the supernatant, enabling comparative analysis of flocculation activity for each compound. Measurements were performed in triplicate. The effect of the solvent (5.9% DMSO) is included as a negative control. (**a**) Compounds **1**–**5**, initially synthesised and screened, (**b**) results for compounds **6**–**8**, synthesised in a second phase of the SAR study.

As shown in Fig. 3, the molecular structure has a significant influence on the observed flocculation activity. The most active compounds (compounds **1** and **3**) induced a rapid and significant drop in optical density within a few minutes, with values remaining relatively constant over time. Notably, the number of methylene groups in the central linker had a pronounced effect on the flocculation efficiency. Compound **2** with an even number of carbon atoms in the central linker appeared less effective. Additionally, increasing the rigidity of the central linker (compound **4**) or positioning the benzimidazole ring in the *meta* position relative to the ether bonds (compound **5**) had a negative impact on flocculation (Fig. 3, a).

Building on the skeleton of the most active compound (compound **1**), we then evaluated the effect of introducing substituents on the benzimidazole rings. These derivatives were synthesised by treating dialdehyde **1a** with the appropriate substituted *o*-phenylenediamine, following previously described conditions. The introduction of substituents significantly influenced flocculation activity, with the most active compounds carrying nitrile (compound **8**) or carboxylic acid (compound **7**) groups, while the presence of a methyl group (compound **6**) on the benzimidazole rings appeared unfavourable (Fig. 3, b).

Although these structural modifications may appear minor, they have a considerable influence on flocculation activity. All synthesised derivatives exhibited flocculation properties, but their efficacy varied depending on specific structural features. Even small changes, such as adding or removing a methylene unit in the linker arm, were found to modulate the activity. These insights highlight several key structural factors enhancing flocculation, including the flexibility of the central linker, the number of methylene groups, and the nature of substituents on the heterocyclic rings. Overall, these observations suggest that the studied phenomenon is highly structure-dependent, with compound **8** emerging as the most potent derivative in the synthesised chemical library. This derivative was therefore selected for mechanistic studies.

### High-resolution microscopy for Floc visualisation

**Fig. 4:**
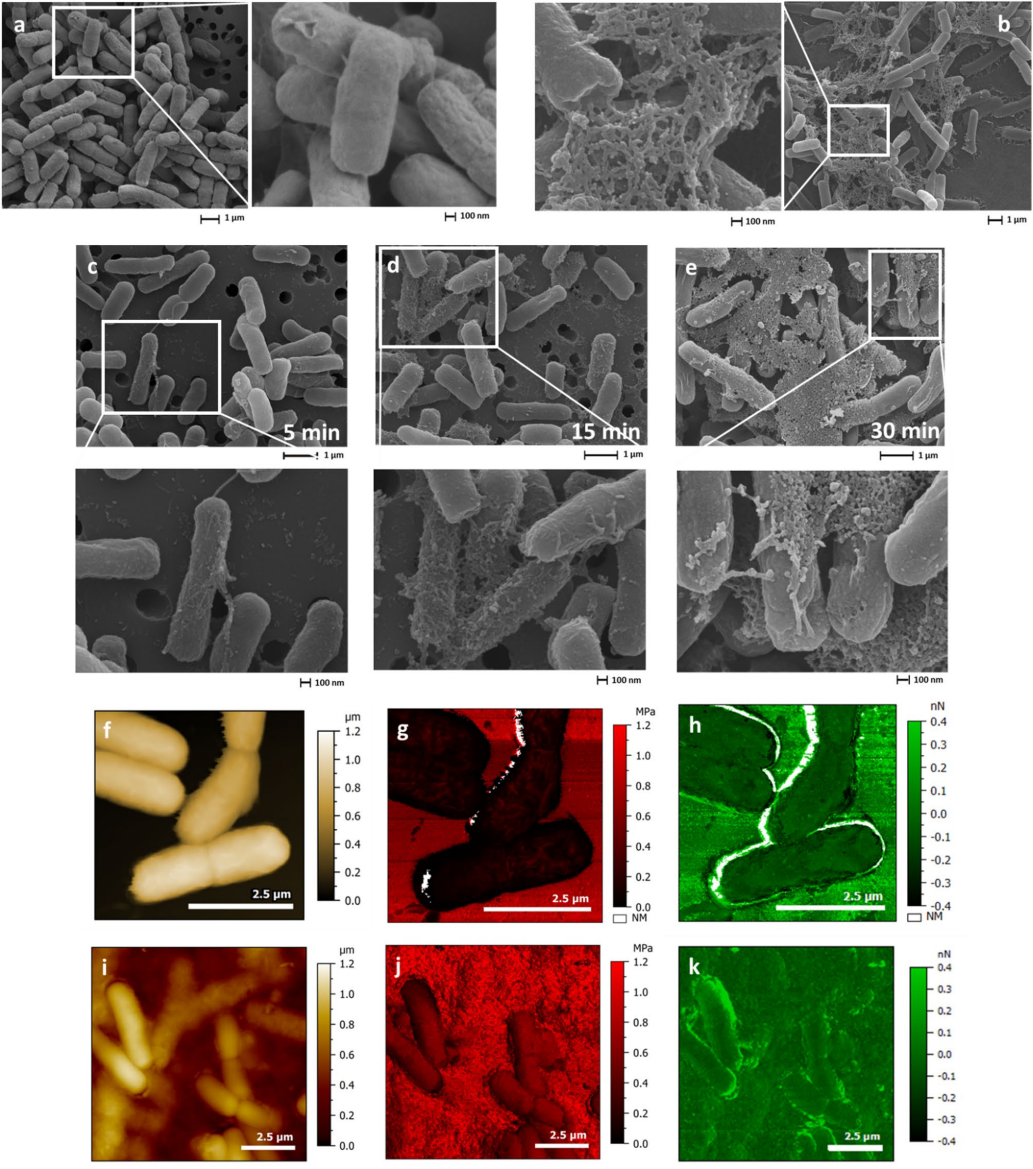
(a–e) Scanning electron microscopy (SEM) images. (a) *E. coli* K-12 MG1655 cells, (b) *E. coli* cells flocculated with compound **8** after 30 min of incubation, (c–e) time-dependent study showing *E. coli* cells incubated with compound **8** for 5, 15, and 30 min, respectively, to assess flocculation progression. (f–k) Atomic force microscopy (AFM) images acquired in fluid (deionised water) using Peak Force Quantitative Nanomechanical (PFQNM) mode. (f–h) Control sample (*E. coli* cells): (f) topography image, (g) modulus map, and (h) adhesion map. (i–k) Flocculated *E. coli* cells: (i) topography image, (j) modulus map, and (k) adhesion map. Areas marked “NM” (non-measured) correspond to pixels where mechanical properties could not be calculated due to insufficient or inconsistent force curve fitting.

Scanning Electron Microscopy (SEM; Fig. 4) was used to assess the impact of compound **8** on *E. coli* morphology over time (5, 15, and 30 min). As expected, unflocculated *E. coli* (strain K-12 MG1655) displayed a typical rod shape with a smooth surface (Fig. 4, a). After 5 min of incubation with compound **8**, vesicle-like protrusions ($\:\pm\:$ 80 nm in diameter) appeared on the bacterial surface, increasing over time and forming a fibrillar web-like network after 30 min (Fig. 4, b–e).

We observed similar microscopic morphologies using Atomic Force Microscopy (AFM) in liquid (i.e., deionised water). Additionally, the mechanical effects of flocculation were assessed using the Peak Force Quantitative Nanomechanical (PFQNM) mode, which provided elastic modulus and adhesion maps for both control and flocculated samples. The elastic modulus of *E. coli* cells (228 ± 61 kPa) aligned with values for other bacteria such as *Staphylococcus aureus* (ATCC 25923)^28^. Some incalculable points, localised at the cell boundary (Fig. 4, g), were identified using a machine learning-based Python home-built script to classify force curves based on their quality (namely good, exploitable, or useless) and cluster mechanical property data. The resulting modulus map, calculated and fitted using Mountains 10.2 software from Digital Surf (Besançon, France) (Fig. S2) revealed two clusters, with *E. coli* stiffness remaining below 300 kPa, lower than that of the poly-L-lysine coated glass substrate (up to 1.2 MPa). Upon exposure to compound **8**, flocculation was observed (Fig. 4, i), accompanied by a significant increase in mechanical rigidity. Compared to control cells (228 ± 61 kPa), flocculated bacteria exhibited a stiffness of 582 ± 90 kPa, with the dense network reaching 830 ± 110 kPa (Fig. 4, j). The adhesion values of *E. coli* and the network remained low, likely due to the tip movement in the aqueous environment or residual cell mobility under tip-induced stress despite prior fixation (Fig. 4, k).

The presence of a fibrillar network within the flocs raised the question of whether flocculation could involve extracellular polymeric substances (EPS) secretion, a well-documented bacterial stress response in Gram-negative bacteria, particularly under antibiotic exposure^29,30^. Moreover, EPS secretion is known to enhance stiffness in biofilms, providing structural support and increased resistance to mechanical stress^31,32^.

### Composition of the Flocs

To assess whether the studied flocculation process follows a conventional pattern (i.e., EPS secretion induced by an external agent^33^, we examined the presence of typical EPS components within the flocs. These generally include extracellular DNA (eDNA), proteins, and polysaccharides, but can also comprise other biomolecules such as RNA and lipids^34^. The presence of DNA was evaluated using a nuclease digestion assay. Specifically, bovine pancreatic deoxyribonuclease I (DNase I) was employed, following a protocol adapted from Ojima et al.^33^. Flocs were collected and treated with DNase I at a concentration of 0.6 mg/mL for 30 min at 37 °C under shaking conditions. As shown in Fig. S3, no noticeable difference was observed in the appearance of the suspension before and after DNase I treatment, indicating that DNA is either absent from the floc matrix or, if present, does not play a significant role in maintaining floc integrity.

The degradation of polysaccharides was investigated using two different methods: cellulase from *Trichoderma reesei*, an enzyme that specifically targets cellulose, and periodic acid, which non-selectively degrades sugars. Following incubation, both methods yielded intact bacterial flocs, suggesting that polysaccharides are not the primary components of the floc matrix. Additionally, quantification of strongly and weakly bound polysaccharides, using a well-established colorimetric method, revealed no significant differences in sugar content between bacterial flocs formed using compound **8** and control *E. coli* samples (Fig. S4).

To investigate the potential proteinaceous nature of the flocs, Proteinase K was applied to bacterial flocs induced by compound **8**. Even after prolonged incubation times, the bacterial flocs retained their integrity. As a complementary technique, AFM combined with infrared spectroscopy (nano-FTIR spectroscopy) was employed. This technique integrates AFM with broadband infrared illumination and FTIR-based detection, enabling high-resolution chemical analysis at the nanoscale ($\:\sim$ 20 nm resolution). Due to its unique capabilities, nano-FTIR spectroscopy has been widely applied in various fields including the characterisation of keratin in hair samples^35^, lipid bilayer analysis in membrane models^36^, and the study of nuclear proteins in lymphocyte cells^37^.

For this study, *E. coli* cultures treated with compound **8** were prepared to induce flocculation, while untreated cultures served as a control. Bacterial cells were deposited on a silicon substrate and air-dried prior to measurement, as current nano-FTIR technology does not allow for high-precision analysis in liquid environments. However, air-dried samples preserve their chemical properties, ensuring reliable spectral interpretation. In addition to topographic imaging, FTIR spectra were acquired at the nanoscale in the 1000–2000 cm^−1^ spectral range.

**Fig. 5:**
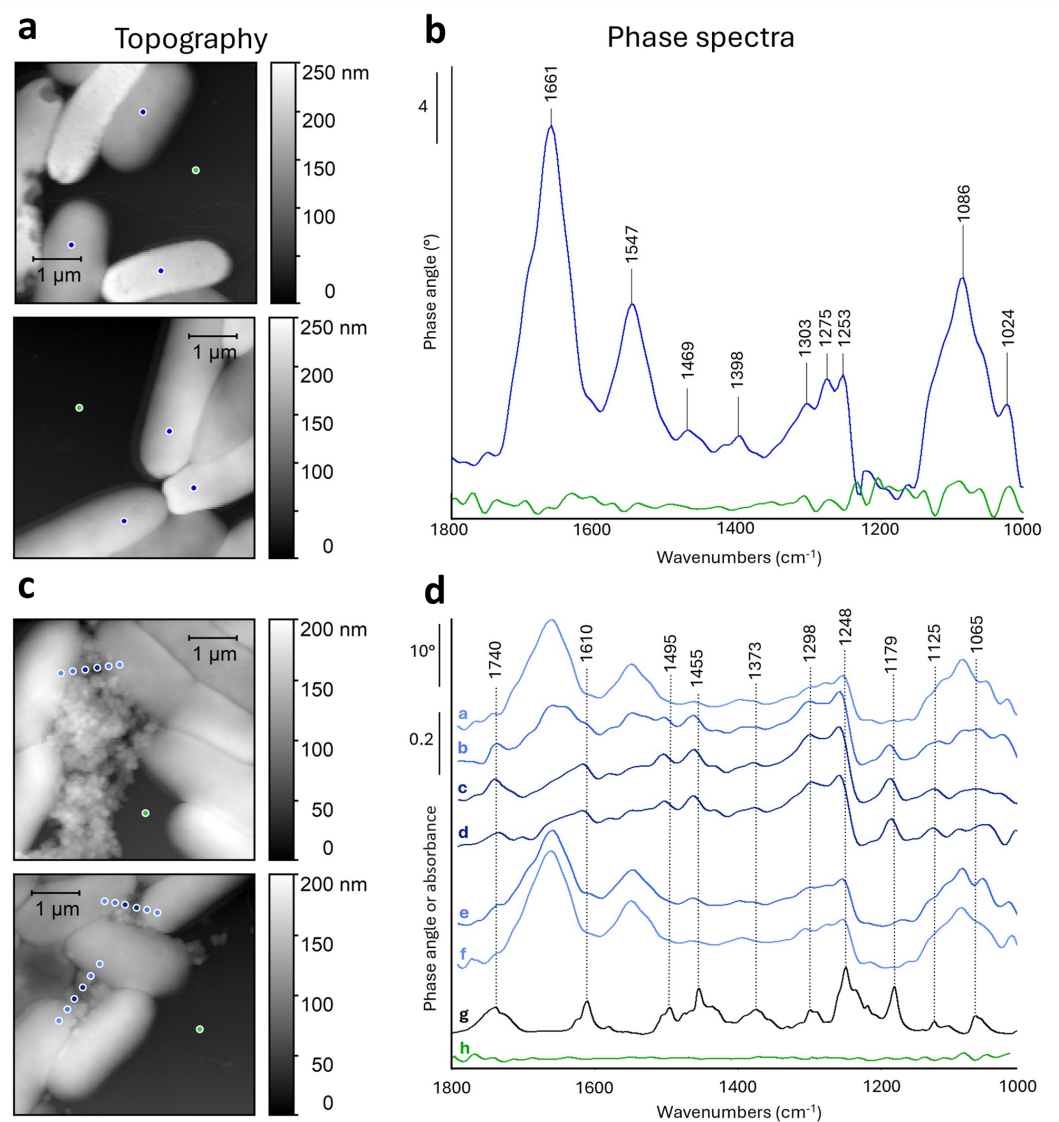
Nano-FTIR spectroscopy of *E. coli* cells incubated with compound **8** for 20 min at 37 °C and of untreated controls. (a) Topography images of untreated *E. coli*. The coloured dots indicate the sample spots where the nano-FTIR spectra were recorded. (b) Average nano-FTIR phase spectrum obtained from untreated *E. coli* compared with the average phase control spectrum from the pure silicon wafer. The *E. coli* spectrum is averaged from six spectra recorded at the positions of the blue dots in panel a. The vertical scale bar on the vertical axis indicates a 4 degrees change in phase angle. (c) Topography images of *E. coli* incubated with compound **8**. The coloured dots indicate again the sample spots where the nano-FTIR spectra were recorded. (d) Averaged phase spectra obtained from the spots indicated in panel c. Each spectrum is an average of three spectra from corresponding spots on the three lines of dots in the two topography images of panel c. Phase spectra a to f correspond to positions from left to right in the topography plots, spectrum h is the average phase control spectrum obtained from the pure silicon wafer, and spectrum g is the FTIR spectrum of compound **8**. The vertical scale bars on the vertical axis indicate a change of 10 degrees in phase angle or of 0.2 in absorbance.

In the control sample, two prominent absorption bands were detected at approximately 1661 cm^− 1^ and 1547 cm^− 1^, corresponding to the amide I and amide II vibrational modes^38^, respectively, indicative of proteinaceous material on the bacterial membrane surface (Fig. 5, a-b). In contrast, analysis of the bacterial flocs (Fig. 5, c-d) revealed distinct spectral variations. Spectra were recorded along three lines spanning from the bacterial surface of one bacterium over the surrounding floc matrix to the surface of the next bacterium. Spectra at corresponding locations along the three lines were averaged: spectra a and f in Fig. 5, d were recorded on the bacteria, spectra b and e close to their edges, and spectra c and d within the floc matrix. The amide I and II bands of proteins are clearly present at the bacterial surface and cell edges, but are drastically reduced within the floc matrix (spectra c and d), indicating that proteinaceous material is largely absent within the flocs.

The use of mutant strains carrying deletions in genes typically associated with exopolysaccharide (EPS) secretion and biofilm formation pathways confirmed the absence of a conventional EPS-dependent flocculation mechanism. The genes considered include members of the *wca* cluster (involved in colanic acid biosynthesis), *pga* (responsible for the synthesis of poly-β-1,6-N-acetyl-D-glucosamine), *csg* (curli fiber production), *yjb* (putatively involved in extracellular polysaccharide export), as well as the *rcs* and *cpx* regulatory systems, which control various stress responses and envelope-associated pathways linked to surface adhesion. As shown in Table 1, mutants defective in these loci still exhibited significant flocculation upon exposure to compound **8**. Notably, all strains were also incubated in the absence of compound **8** as a negative control, and none of them showed any flocculation under these conditions, confirming that flocculation was specifically induced by compound **8**. A detailed description of the genetic deletions and their associated functions can be found in the Supporting Information (Table S1).

**Table 1:** Response of the various *E. coli* mutants considered for the flocculation efficiency studies involving compound **8**.

Mutants	Flocculation efficiency
BW25113	+++
BW25113 ∆wcaA/∆wcaB/∆wcaC/∆wcaD	+++
BW25113 ∆pgaA/∆pgaB/∆pgaC/∆pgaD	+++
BW25113 ∆csgA/∆csgB/∆csgC/∆csgD	+++
BW25113 ∆yjbE/∆yjbF/∆yjbG/∆yjbH	+++
MG1655	+++
MG1655 ∆rcs	+++
MG1655 ∆cpx	+++
MG1655 AgO^+^	+

The flocculation efficiency is summarised using the following scale: high flocculation (+++), medium flocculation (++), low flocculation (+), and no flocculation (−).

**Fig. 6:**
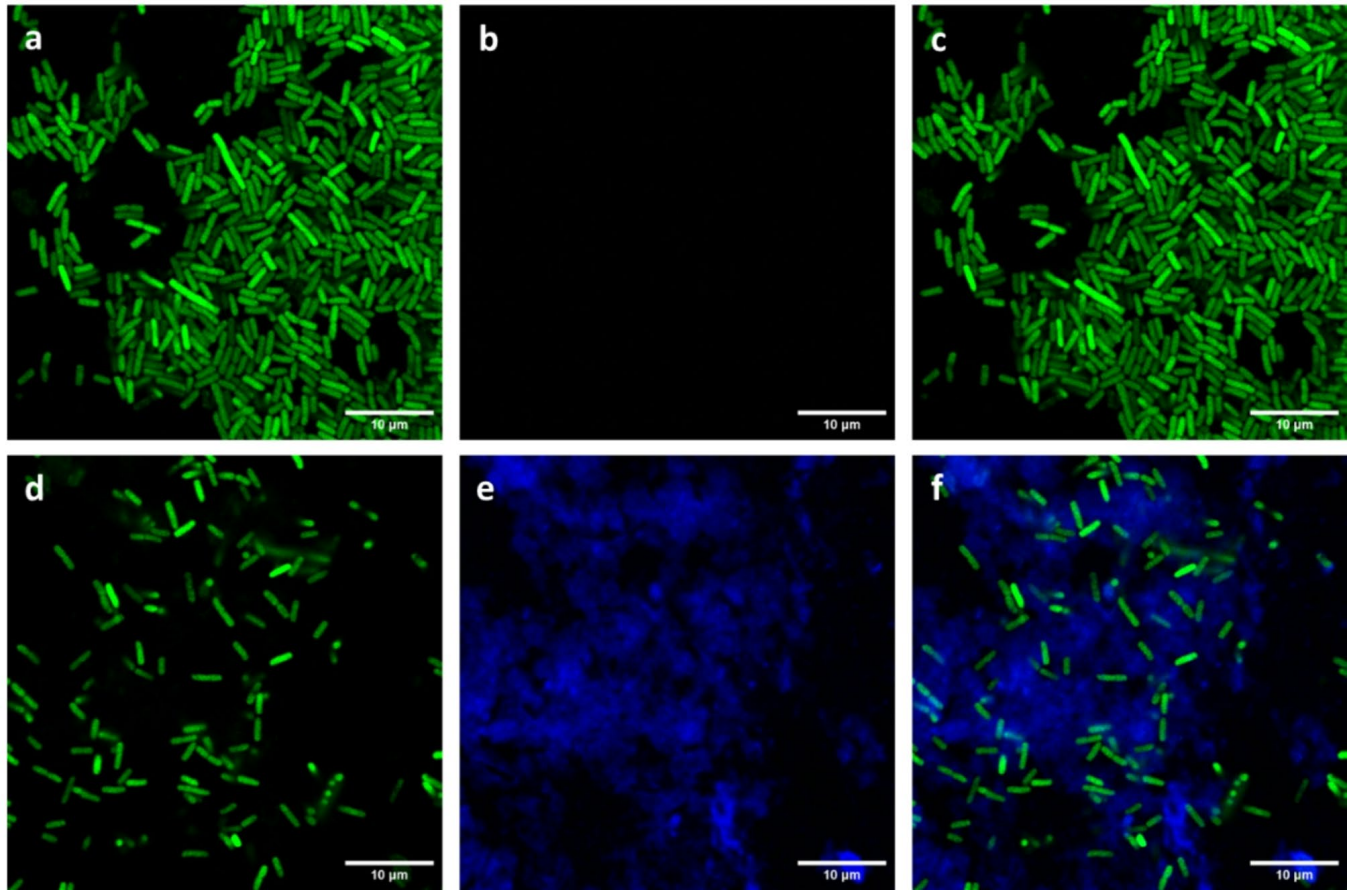
Confocal microscopy images of fluorescent *E. coli* cultures. Panels (**a**–**c**) show a genetically modified *E. coli* K-12 MG1655 strain expressing green fluorescent protein (GFP; λ_em_ = 509 nm), observed respectively using (**a**) the green channel, (**b**) the blue channel, and (**c**) a merge of both. Panels (**d**–**f**) show a culture of the same *E. coli* strain after flocculation with compound **8**, observed under the same conditions: (**d**) green channel, (**e**) blue channel, and (**f**) merged image. The green signal corresponds to fluorescence of the bacterial GFP, while the blue signal corresponds to the intrinsic fluorescence of compound **8** (λ_ex_ = 318 nm, (λ_em_ = 376 nm). Scale bar: 10 μm.

Interestingly, nano-FTIR measurements allowed us to reveal the spectral features of the floc matrix, which were observed at 1738, 1617, 1578, 1503, 1461, 1432, 1377, 1297, 1258, 1185, 1124, and 1051 cm^−1^ (in the average of spectra c and d of Fig. 5, panel d). These bands nearly exactly match the characteristic absorption peaks in the FTIR spectrum of compound **8** (Fig. 5d, spectrum g), suggesting that it is a main constituent of the floc matrix. Some of the nano-FTIR bands are upshifted by a few cm^− 1^ relative to those of the macroscopic FTIR spectrum, which is a known phenomenon^39^. Confocal microscopy (Fig. 6) confirmed the presence of compound **8** in bacterial flocs by exploiting its intrinsic fluorescence (λ_ex_ = 318 nm, λ_em_ = 376 nm). A GFP-expressing *E. coli* K-12 MG1655 strain (λ_em_ = 509 nm) allowed the differentiation between bacterial cells and the compound. While control samples showed only green fluorescence, flocculated bacteria exhibited a blue-emitting halo around the clusters, indicating that compound **8** was integrated into the floc structure.

### Mechanistic insights into flocculation

Peptides containing tryptophan motifs have been shown to self-assemble into molecular fibres, as demonstrated by Zhang et al.^40^. Interestingly, these self-assembled peptides exhibit a strong affinity for bacterial membranes, leading to *E. coli* flocculation through a mechanism distinct from conventional pathways. Similarly, benzimidazole derivatives are also known to undergo molecular self-assembly. Dhinakaran et al. reported that amphiphilic *N*-glycosylamines based on partially protected benzimidazole structures form molecular fibres and supramolecular gels in various solvents^41^. More recently, Srideep et al. demonstrated that benzoperylene benzimidazoles (BPBIs) can self-assemble into nanoribbons, nanorods, nanofibres, and nanoparticles, depending on the solvent^42^.

From a structural point of view, compound **8** enables multiple non-covalent interactions, including π-type interactions, hydrogen bonding, and dipole-dipole interactions, particularly involving polar groups like nitriles. These interactions promote self-assembly and the formation of supramolecular structures.

**Fig. 7:**
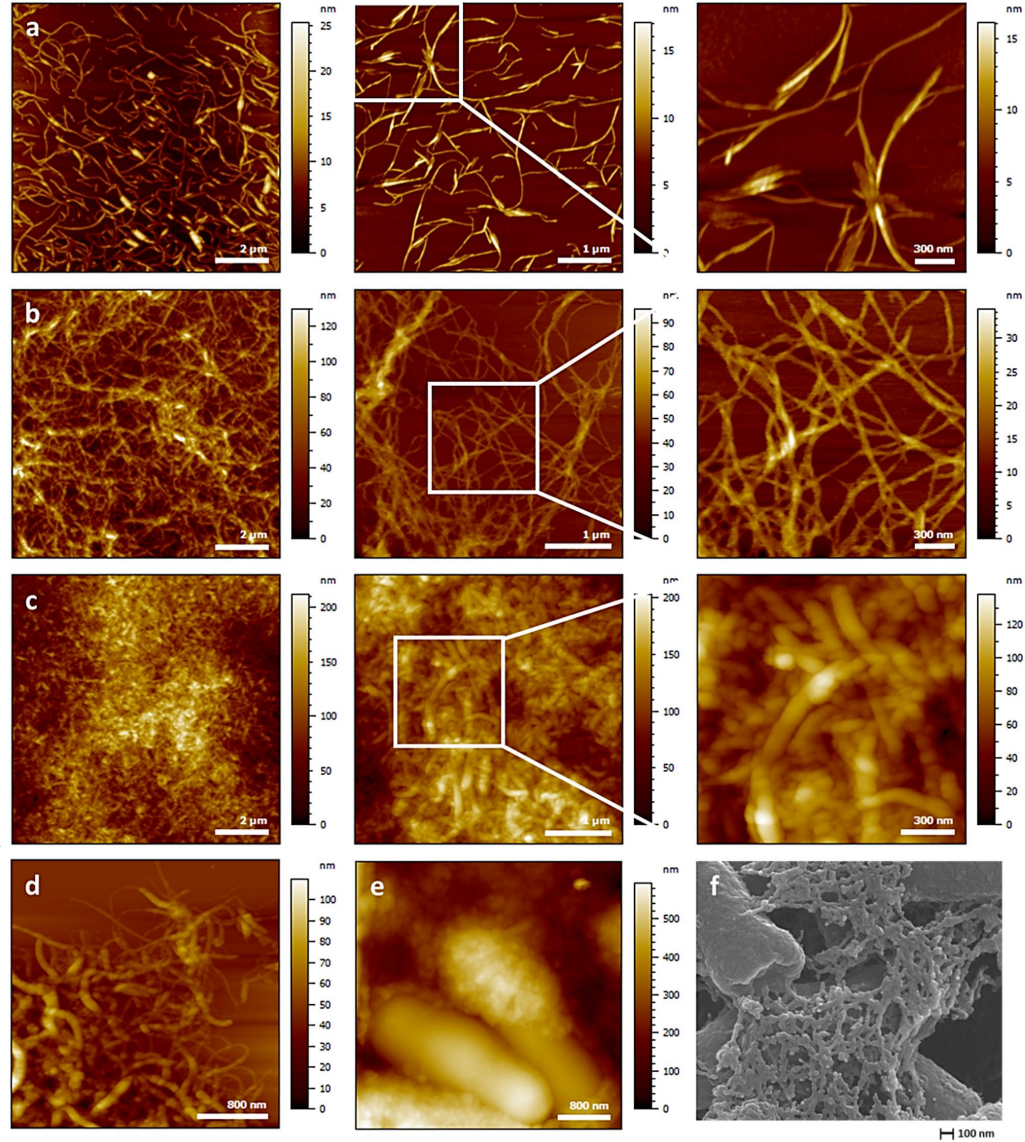
Atomic force microscopy (AFM) images of compound **8** thin films prepared by dip-coating. Images were taken in tapping mode in air. (**a**) Thin film on mica, DMSO solution, (**b**) thin film on silicon wafer, DMSO solution and (**c**) thin film on mica, H_2_O/DMSO solution (5.9% DMSO). (**d**) Self-assembled fibres on mica, H_2_O/DMSO solution (5.9% DMSO), imaged in tapping mode in air, (**e**) *E. coli* flocs observed in liquid using PeakForce QNM mode, (**f**) *E. coli* flocs imaged by scanning electron microscopy (SEM).

To assess the ability of compound **8** to self-assemble, molecular thin deposits were prepared using the dip-coating method on mica and silicon wafers. AFM analysis of the dried films revealed the formation of molecular fibres on both substrates, confirming the self-assembling behaviour of compound **8** (Fig. 7, a-b). While the fibres appeared more interconnected on silicon, their width (50 $\:\pm\:$ 5 nm on mica vs. 53 $\:\pm\:$ 5 nm on silicon) and thickness (5 $\:\pm\:$ 1 nm on mica vs. 9 $\:\pm\:$ 1 nm on silicon) were comparable, indicating that fibre formation is largely independent of the substrate nature.

An additional deposit was prepared on mica under conditions that closely mimic the experimental flocculation conditions (i.e., compound **8** concentration of 0.03 mg/mL in an H_2_O/DMSO mixture containing 5.9% DMSO). AFM analysis (Fig. 7, c) revealed a denser fibrillar network with increased fibre thickness, exhibiting heights (20 $\:\pm\:$ 1 nm) and widths (86 $\:\pm\:$ 17 nm) nearly twice those observed under initial conditions. Notably, these fibres exhibit morphological similarities with those observed within the flocs by SEM and fluid AFM, revealing grain-like structures assembled into a dense, highly interconnected fibrous network (Fig. 7, d–f). These findings suggest that the self-assembly of compound **8** into supramolecular fibres plays a key role in *E. coli* flocculation, forming the primary structural component of the flocs.

While it is proposed that compound **8** induces bacterial flocculation through a mechanism involving self-assembled fibres network formation, our findings indicate that flocculation efficiency is influenced by the bacterial strain used. Notably, we observed that an *E. coli* K-12 mutant with a restored *O*-antigen exhibited significantly reduced flocculation efficiency compared to the wild-type strain (Table S1 and Table 1). The *O*-antigen, among other functions, acts as a protective barrier that enhances resistance by restricting the entry of external molecules^43^. These findings suggest a potential role for direct interactions between compound **8** and the bacterial envelope, or possibly the membrane, in the flocculation process.

**Fig. 8:**
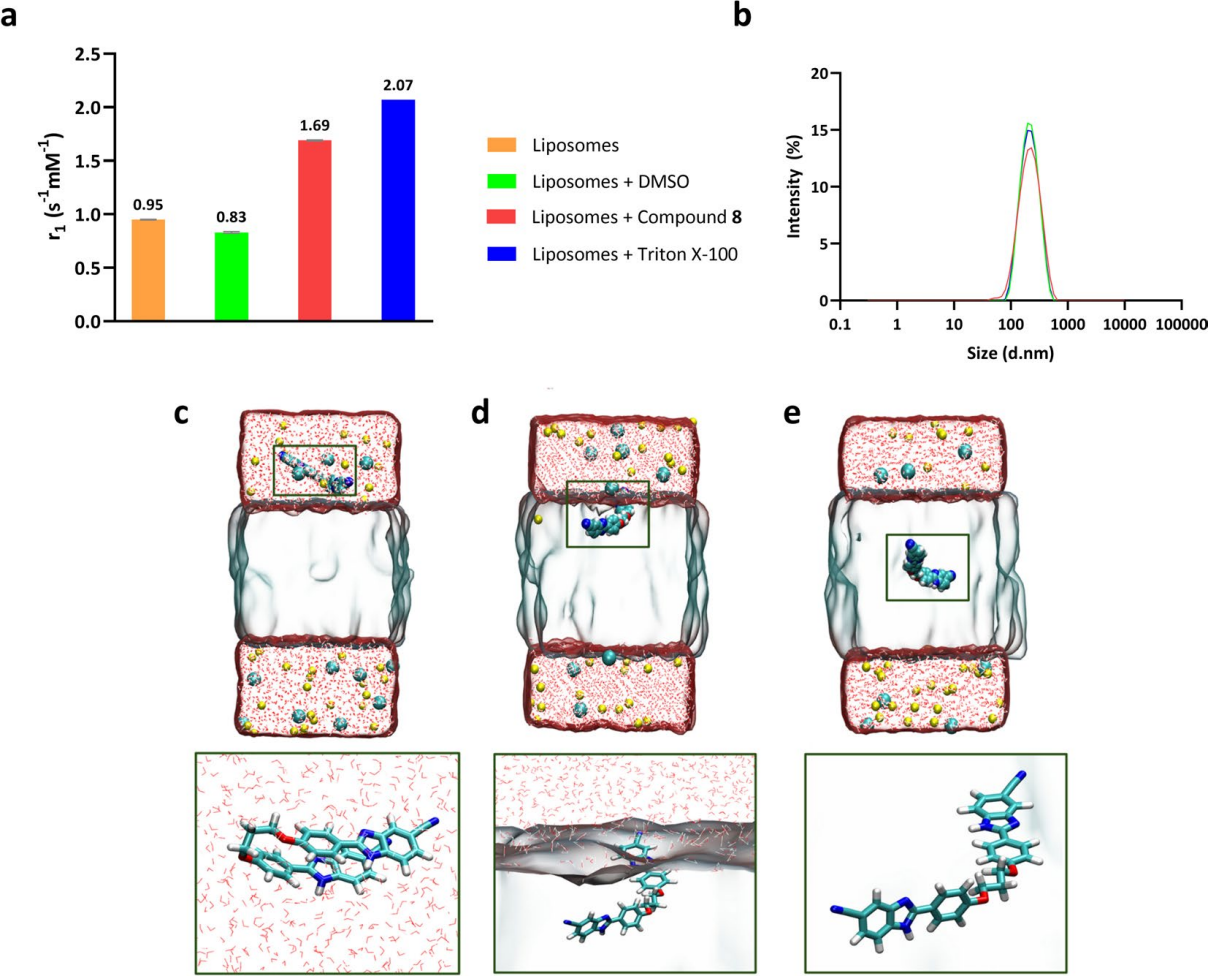
(**a**) Longitudinal relaxivity values (r_1_, 60 MHz, 37 °C) measured for liposomes in the presence of DMSO (negative control), compound **8** (59 µM), and Triton X-100 (positive control) under conditions of [PO_4_^3−^] = 2.8 mM and a compound-to-lipid ratio of ≅ 1:50. (**b**) Dynamic light scattering (DLS) analysis of paramagnetic liposomal solution after 30 min of treatment with 59 µM of compound **8**. (**c**–**e**) Snapshots from molecular dynamics simulations of a DPPC/DPPG membrane model in the presence of compound **8**, where the ligand was initially positioned (**c**) in the aqueous medium (system (**I**)), (**d**) at the aqueous medium-lipid bilayer interface (system (**II**)), and (**e**) within the lipid bilayer (system (**III**)).

Paramagnetic liposomes (DPPC/DPPG, 7:3) encapsulating Gd-HPDO3A were used to assess the membrane interaction of compound **8**. The relaxivity of control liposomes was 0.950 ± 0.004 s⁻¹mM⁻¹, which slightly decreased with DMSO (5.9%) (possibly due to increased viscosity). Incubation with the derivative (59 µM, 30 min) resulted in a twofold increase in relaxivity, indicating membrane interaction, though less than the positive control (Triton X-100) (Fig. 8, a). Dynamic light scattering (DLS) showed that liposome integrity was maintained post-treatment, suggesting the derivative alters membrane fluidity, as inferred from the relaxometric data, without causing pore formation or disrupting the bilayer (Fig. 8, b). To confirm this observation, the integrity of the *E. coli* membrane was assessed using 8-anilinonaphthalene-1-sulfonic acid (ANS) for the outer membrane and 3,3’-dipropylthiadicarbocyanine iodide (DiSC₃(5)) for the inner membrane. No significant disruption, such as pore formation, was detected under these conditions (Fig. S5). These findings confirm that compound **8** can interact with bacterial membranes, but through an anchoring mechanism rather than inducing membrane damage.

In addition to experimental studies, molecular dynamics (MD) simulations were performed to assess the membrane interaction properties of the compound using a simplified DPPC/DPPG bilayer model (Fig. 8, c–e). Three systems were designed with different initial placements of the derivative: (**I**) fully immersed in the aqueous phase, 30 Å from the bilayer; (**II**) at the interface near the polar heads; and (**III**) embedded within the bilayer upper hydrophobic region. Each simulation lasted 1 µs with a 4 fs timestep.

The trajectory analysis provided the following insights. In system (**I**), the molecule exhibited pronounced instability, adopting a folded conformation to optimise hydrophobic interactions and minimise interactions with aqueous medium (Fig. 8, c). Interestingly, observations revealed a tendency for the derivative to unfold, particularly upon approaching the lipid bilayer and engaging with its polar heads. Within system (**II**), rapid movements towards the membrane compartment occurred shortly after the onset of dynamics (within a few ns). Upon integration into the lipid bilayer, the derivative assumed a relatively stable “V-shaped” structure over simulation time (Fig. 8, d). In system (**III**), the ligand maintained a relatively stable conformation throughout the molecular dynamics, adopting a slightly filled (“V-shaped”) arrangement (Fig. 8, e). However, the derivative exhibited numerous translational movements in the upper part of the bilayer during the simulation.

The ligand RMSD was also calculated along the simulation, to gain insights into the dynamic behaviour of the molecule (Fig. S6). In system (**I**), the RMSD values fluctuated around 6.5 Å with periodic spikes of $\:\sim$ 3 Å. These fluctuations indicate significant changes in the ligand conformation during the simulation. Initially positioned in the bulk water, the ligand tends to move closer to the lipid bilayer, reflected by higher RMSD values, and occasionally returns to the bulk phase, leading to lower RMSD values. In system (**II**), the RMSD analysis supports the tendency of the ligand to insert into the lipid bilayer and maintain a stable conformation. This stability suggests a strong interaction between the ligand and the lipid environment, indicating potential membrane integration. Similarly, in system (**III**), the RMSD analysis indicates a consistent and stable conformation, underscoring the favourable interaction and integration of the ligand within the lipid bilayer. In both systems (**II**) and (**III**), several RMSD peaks around ~ 3 Å suggest minor fluctuations due to water molecules penetrating the lipid bilayer when the ligand approaches bulk water. The ligand then retreats into the hydrophobic membrane environment, highlighting its preference for lipid interactions and its overall stability within the membrane.

Based on these results, we propose that flocculation induced by benzimidazole derivatives operates through a bridging mechanism. In the case of EPS secretion, flocculation typically arises from the formation of a fibrillar network composed of extracellular polymeric substances secreted by the bacteria, which mediate cell bridging. In contrast, with benzimidazole derivatives, the fibres result from an external process of molecular self-assembly. These interactions occur at the bacterial surface, where the molecules, either as monomers or possibly as protofibrils, anchor into the bacterial membrane. This anchoring serves as a nucleation site for the formation of a fibrillar network, ultimately promoting the bridging of bacterial cells and leading to flocculation.

**Fig. 9:**
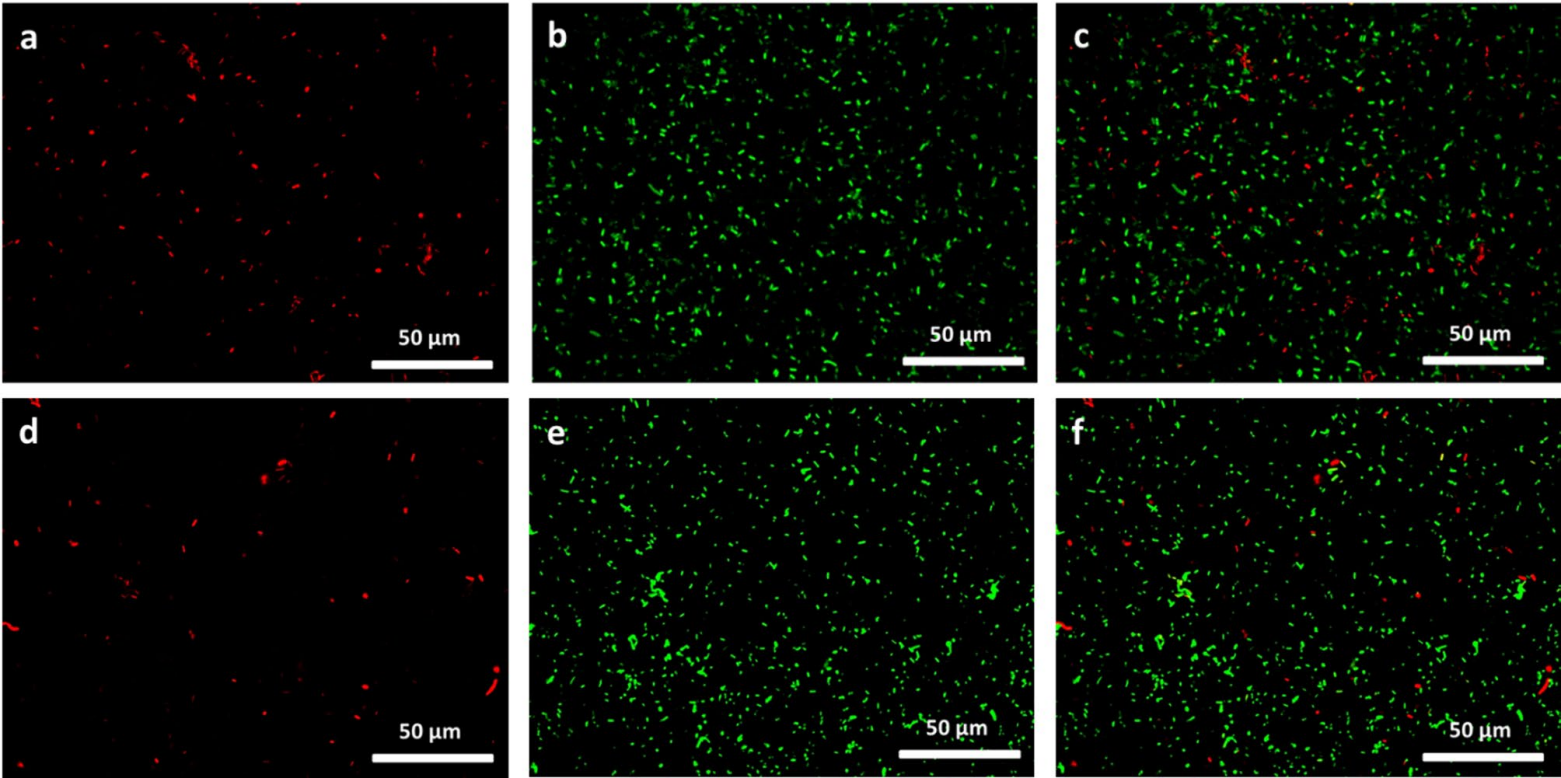
Fluorescence microscopy images of *E. coli* stained with the Live/Dead BacLight^®^ viability kit to assess bacterial viability. Panels a-c show control bacteria incubated with 5.9% DMSO (control), while panels d-f show *E. coli* flocculated with compound **8** for 30 min. Green fluorescence (SYTO 9) indicates live cells, and red fluorescence (propidium iodide) marks death cells. Images a and d display the green channel (live), b and e the red channel (dead), and c and f the merged channels. Both conditions exhibit comparable viability levels, indicating that compound **8** does not induce excessive mortality.

To assess bacterial viability following flocculation, the Live/Dead BacLight^®^ Bacterial Viability kit was used. No significant difference was observed between the DMSO control and the sample treated with compound **8**, with both showing approximately 70 ± 1% viability (Fig. 9), indicating that compound **8** does not induce substantial bacterial mortality. This was further supported by the presence of numerous colonies after plating both the flocs and supernatants on soft agar (Fig. S7). To assess the broader applicability of compound **8**, its flocculation activity was tested on four additional bacterial strains: two Gram-negative (*Phyllobacterium myrsinacearum* ATCC 43591, *Cupriavidus metallidurans* ATCC 43123) and two Gram-positive (*Micrococcus luteus* ATCC 4698, *Lactobacillus rhamnosus* ATCC 7469). All strains showed substantial flocculation, with efficiencies ranging from ~ 52–73% (Fig. S8). These findings lead to two key conclusions. First, the *O*-antigen barrier is not absolute: both Gram-negative strains possess LPS yet remain susceptible, suggesting their *O*-antigens may be shorter or less densely packed. This highlights that flocculation efficiency depends more on *O*-antigen structural features than on its mere presence. Second, the activity observed in Gram-positive strains confirms that the mechanism is not restricted by Gram classification, expanding its potential use across diverse bacterial taxa. Collectively, these results underscore the promise of this flocculation strategy for biotechnological applications, including bacterial immobilisation.

## Conclusions

This study provides new insights into the flocculation mechanism of bisbenzimidazole derivatives, revealing a self-assembly-driven process distinct from classical electrostatic or EPS-mediated pathways. We demonstrated that these derivatives rapidly induce *E. coli* aggregation (within 10 min) at low micromolar concentrations (59 µM), with SAR analysis identifying key structural features essential for flocculation activity, confirming the structure-dependent nature of the process. High-resolution microscopy (SEM, fluid AFM) revealed a dense fibrillar network within bacterial flocs, initially resembling extracellular polymeric substances (EPS). However, further investigations ruled out EPS secretion, with nano-FTIR spectroscopy and confocal microscopy confirming that the flocs primarily consist of the bisbenzimidazole derivative. This led to the investigation of the derivative’s ability to self-assemble into fibres, supporting a flocculation mechanism driven by supramolecular self-assembly. Variations in flocculation efficiency across bacterial strains suggested the involvement of additional factors. In particular, the presence of *O*-antigen on the bacterial outer membrane significantly reduced flocculation, pointing to a membrane interaction component. Experimental and in silico studies confirmed that the derivative preferentially interacts with the hydrophobic compartment of the membrane without compromising its integrity. Based on these findings, we propose a bridging mechanism whereby the bisbenzimidazole derivative anchors to bacterial membranes, acting as a nucleation site for the formation of an interconnected network of self-assembled fibres, thereby promoting flocculation. By elucidating the dual role of bisbenzimidazole derivatives in membrane anchoring and fibre formation, this study advances our understanding of their unique flocculation mechanism. This work could therefore be helpful for biotechnological applications, particularly in bacterial immobilisation and industrial flocculation processes, paving the way for future developments.

## Experimental section

### Chemistry

The ^1^H NMR analyses were carried out at room temperature (25 °C), in 5 mm diameter tubes using deuterated solvents (mainly DMSO-d_6_) using a Bruker^®^ Avance NEO (600 MHz). Chemical shifts are expressed in ppm relative to tetramethylsilane (TMS) used as an internal reference (0.00 ppm), present in the deuterated solvents used. Infrared spectroscopy analyses were carried out on solid-phase samples in the spectral range 650–4000 cm^− 1^ using a Perkin Elmer Spectrum 100 spectrometer. Mass spectrometry analyses (Waters QToF API-US, electrospray source (ESI)) were performed in positive mode using stock solutions in DMSO (5 mg/mL) diluted 1000 x in acetonitrile before injection at a flow rate of 5 µL/min. The ESI conditions used were: 3.1 kV potential on the capillary, 30 V potential on the cone, a source temperature of 80 °C and a desolvation temperature of 120 °C. Solvents and reagents were commercially available derivatives (Aldrich, Alfa Aesar, Acros Organics, BLDPharm, Fisher Scientific) and were used without prior purification.

#### Preparation of the bisbenzaldehyde compounds

The bisbenzaldehydes (**1–5a**) were prepared by reaction between 4-hydroxybenzaldehyde (21 mmol) and a solution of corresponding α,ω-dibromoalkane (10 mmol) in ethanol (6 mL) in the presence of sodium carbonate (10 mmol). The reaction mixture was then refluxed overnight. After cooling, the resulting precipitate was filtered, and the solid was washed sequentially with water and ethanol. Compound **4a** was prepared in the same way, using a solution of m-xylylene dibromide instead of dibromoalkanes. Non-optimised yields ranged from 45 to 90%, with bisbenzaldehydes of sufficiently high purity to proceed to a second step.

All bisbenzaldehydes are already described in the literature: **1a–3a**^44^ and **4a**^45^, **5a**^46^.

#### Preparation of the bisbenzimidazole compounds

The corresponding benzimidazoles were thus prepared by adding a (non)-substituted *o*-phenylene diamine derivative. Bisbenzaldehyde derivative (1 mmol) was added to a solution of *o*-phenylene diamine derivative (2.2 mmol) in a 50:50 ethanol/water mixture, along with sodium metabilsulfite (2.2 mmol). The reaction mixture was refluxed overnight. After cooling, the resulting precipitate was filtered and washed sequentially with water and ethanol (or methanol in some cases).

The majority of the bisbenzimidazoles considered are described in the literature: **1–3**^27^, **4–5**^47^, **6–7**^48^.

##### 2,2′-((propane-1,3-diylbis(oxy))bis(4,1-phenylene))bis(1 H-benzo[d]imidazole-5-carbonitrile) (**8**)

Yield (not optimised): 78%, ^1^H NMR (DMSO-*d*_*6*_, 600 MHz): δ 2.27 (q, 2 H, *J* = 6.2 Hz), 4.28 (t, 4 H, *J* = 6.2 Hz), 7.19 (d, 4 H, *J* = 8.9 Hz), 7.57 (d, 2 H, *J* = 8.3 Hz), 7.71 (d, 2 H, *J* = 8.3 Hz), 8.08 (s, 2 H), 8.16 (d, 4 H, *J* = 8.8 Hz); ^13^C{^1^H} (DMSO-*d*_*6*_, 600 MHz): δ 29.0, 65.0, 104.1, 115.5, 120.6, 122.1, 129.1, 161.0; IR: 3500 (N-H), 2240 (C ≡ N), 1600 (C = C aromatic), 1250, 1060 (C-O) cm^− 1^; HRMS (ESI^+^) calculated for C_31_H_22_N_6_O_2_ *m/z* 511.1883 [M + H]^+^, found 511.1882 [M + H]^+^ (mass deviation [M + H]^+^ = 0.2 ppm).

Spectroscopic characterisation of compound **8** is provided in the Supporting Information, including ¹H NMR (Fig. S9), ¹³C NMR (Fig. S10), and HRMS data (Fig. S11).

#### Thin films preparation

For the dip coating method, the initial samples were prepared from a solution of compound **8** at a concentration of 0.2 mg/mL. A substrate suitable for AFM studies, either a 1 cm^2^ mica square (Ted Pella^®^, Inc.) or a 1 cm^2^ silicon substrate (silicon wafer type P/[100], Ted Pella^®^, Inc.), was immersed in this solution for 1 h. The substrate was then removed from the solution and allowed to dry in a fume hood until the solvent had completely evaporated (12 h). Following the same protocol, additional samples were prepared using a solution containing a H_2_O/DMSO mixture (5.9% DMSO) and a compound concentration of 59 µM, with mica substrates (1 cm^2^ mica square, Ted Pella^®^, Inc.).

AFM measurements on the different thin-film samples were performed at room temperature using a Dimension Icon AFM (Bruker^®^, Santa Barbara, CA, USA). The measurements were conducted in air using tapping mode. A RTESPA-150-30 probe (Bruker^®^ AFM Probes, Camarillo, CA, USA) with a pre-calibrated spring constant of 5 N/m and a tip radius of 30 nm was used. Each image was scanned at a resolution of 256 $\:\times\:$ 256 points. The measurements were carried out with Nanoscope software Version 9.7, and data analysis, including height and width measurements, was completed using Nanoscope Analysis Version 2.0.

### Biological assays

#### Bacterial culture

Bacterial cultures of *Escherichia coli* (K-12 MG1655, ATTC 47076, sourced from BCCM culture collection) were prepared under standard conditions, in a LB medium supplemented with glucose (10 g tryptone, 5 g yeast extract, 5 g NaCl and 1 g glucose in 1 L Milli-Q water). An aliquot of the *E. coli* K-12 MG1655 strain preserved in a cryoprotectant (culture medium with 25% glycerol) and stored at -80 °C was used to generate a preculture. Thawed aliquots were sub-cultured onto a solid medium (LB supplemented with 15% of bacteriological agar, VWR), and bacteria were incubated for 24 h to resume growth. An isolated colony was then transferred to a liquid culture medium and incubated at 37 °C. Growth was monitored by measuring the optical density (O.D.) at 600 nm using a S800 Diode Array Spectrophotometer, Biochrom^®^. Incubation was pursued until the O.D. reached approximately 0.6 (1.5 × 10^8^ CFU/mL). The working culture was then prepared by transferring a volume of the preculture into fresh liquid medium and incubated at 37 °C with agitation until the desired O.D. for the designed experiment was reached.

*Lactobacillus rhamnosus* (ATCC 7469), *Micrococcus luteus* (ATCC 4698), *Cupriavidus metallidurans* (ATCC 43123), and *Phyllobacterium myrsinacearum* (ATCC 43591) were additionally included in this study and cultured under the same conditions as described above.

#### Flocculation activity

Flocculation activity evaluation was carried out using a bacterial culture with an O.D. of 0.3–0.4. The tested compounds were dissolved in DMSO. Each solution was then added to a sterile 15 mL tube (Falcon^®^, VWR) containing the bacterial culture, achieving a final compound concentration of 59 µM (DMSO percentage, 5.9%). After vortexing, the samples were incubated at 37 °C and 150 rpm using a INFORS HT^®^ Aquatron. Absorbance measurements of the supernatant (600 nm) were taken at 0, 10, and 30 min after exposure to the evaluated compound using a S800 Diode Array Spectrophotometer, Biochrom^®^. Between each measurement, the samples were kept at 37 °C to allow for continuous bacterial growth. All measurements were performed in triplicate for each compound, and the average of three runs was designated as the final value. A negative control consisting of 5.9% DMSO was also included in the evaluation.

Optical densities were then normalised as follows (Eq. (1)):

1 $$\:\varvec{B}\varvec{a}\varvec{c}\varvec{t}\varvec{e}\varvec{r}\varvec{i}\varvec{a}\varvec{l}\:\varvec{p}\varvec{o}\varvec{p}\varvec{u}\varvec{l}\varvec{a}\varvec{t}\varvec{i}\varvec{o}\varvec{n}\:\left(\varvec{\%}\right)\:=\:\frac{\varvec{O}.\varvec{D}\:\left(\varvec{t}\right)}{\varvec{O}.\varvec{D}\:\left({\varvec{t}}_{0}\right)}\:\times\:100$$

O.D. (t) = optical density measured at time t.

O.D. (t_0_) = optical density measured at time 0 min (initial).

#### Microscopic approaches

##### Scanning electron microscopy

A bacterial culture with an O.D. of 0.5 was incubated for 30 min at 37 °C in the presence of either 59 µM of compound **8** or 5.9% DMSO (control). After incubation, the samples were filtered through a 0.4 μm Millipore^®^ filter to deposit the cells on the filter surface. Each sample was then fixed by immersion in a non-acetic Bouin solution (75 mL picric acid-saturated solution and 20 mL formalin) for two hours. Following fixation, the samples were dehydrated by sequentially immersing them in ethanol baths of increasing concentrations, ranging from 70 to 100%. The standard chemical drying technique was then applied, involving ethanol and HMDS (hexamethyldisilazane) baths with progressively increasing HMDS concentrations. After evaporation of the final drying bath, the samples were sputter-coated with a thin layer of gold/palladium. Observations were conducted either using a JEOL JSM-7200 F with secondary electron imaging at 1.50 kV and a working distance of 6.4 mm, or a FEG-ESEM QUANTA F200 (FEI) in high vacuum mode with secondary electron imaging at 30 kV and a working distance of 8.8 mm.

##### Atomic force microscopy

A bacterial culture with an O.D. of 0.5 was incubated for 30 min at 37 °C with compound **8** at a concentration of 59 µM or used directly as a control. The samples were then centrifuged for 10 min (at 3000 rpm for the control or 1500 rpm for flocculated bacteria, using a Thermo Scientific^®^ IEC CL31R Multispeed) and the resulting pellet was washed with Milli-Q water. After washing, each sample was fixed with a paraformaldehyde solution (4% in PBS, Thermo Fisher^®^) for 15 min at room temperature. The solution was then removed, and each pellet was washed with Milli-Q water again. The pellets were resuspended in a small volume of Milli-Q water (300 µL for the control and 800 µL for flocculated bacteria), and 20 µL of each suspension was placed on a poly-L-lysine-coated glass slide within a 25 µL gene frame (Thermo Fisher^®^). The glass slides were prepared by ultrasonically cleaning them with 70% ethanol, then drying and applying a 100 µL drop of commercial poly-L-lysine solution (M.W. 150000–300000 0.1% (w/v) in water, Sigma Aldrich), and allowing them to dry in a laminar flow hood. After drying for a few minutes, the samples were gently rinsed with Milli-Q water, and 100 µL of solvent was added to the gene frame for liquid medium observations.

Measurements were conducted in liquid using the PeakForce QNM (PFQNM) mode (Bruker^®^). A PFQNM-LC-A-CAL probe (Bruker^®^ AFM Probes, Camarillo, CA, USA) was used for measurements in liquid, featuring a short paddle-shaped cantilever with a pre-calibrated spring constant of approximately 0.1 N m⁻¹, a resonance frequency of around 45 kHz, a tip radius of 65 nm, and a tip length of 17 μm. An applied force of 1.5 nN was used to ensure good contact between the cells and the tip without causing any damage to the cells. The tip-sample distance was set to a range of 400–800 μm to accommodate the cell height in liquid. The value obtained was calculated as the average value from between 10 and 20 bacteria. Each image was scanned at 256 $\:\times\:$ 256 points, which, in the case of PFQNM analysis, generated 65,536-pixel force-separation curves.

All AFM measurements were conducted at room temperature using a Dimension Icon AFM from Bruker^®^ (Santa Barbara, CA, USA). The measurements were executed with Nanoscope software Version 9.7, while data acquisition was carried out using Nanoscope Analysis Version 2.0 and the Python code developed at LPNE named PyCAROS (Python Code for Approach Retract curve analysis on Organic and Soft materials). Image analysis was carried out on Mountains 10.2 software from Digital Surf (Besançon, France). For statistical analysis, including Gaussian fitting of the collected mechanical data, Origin 2018 was employed.

##### Nano-FTIR measurements

A bacterial culture with an O.D. of 0.5 was centrifuged at 3000 rpm for 5 min to remove the culture medium, and the pellet was washed with PBS. The culture was either left as a control or incubated with 59 µM of compound **8** at 37 °C for 20 min to induce flocculation. After incubation, all samples were centrifuged (3000 rpm for control and 1500 rpm for flocculated bacteria) for 5 min, and the pellets were washed with Milli-Q water. The washed pellets were resuspended in 300 µL of Milli-Q water for the control and 800 µL for the flocculated bacteria. Twenty microliters of each suspension were deposited on silicon wafers suitable for nano-FTIR spectroscopy (silicon wafer type P/[100], Ted Pella^®^, Inc.) and allowed to dry completely in a fume hood.

Nanoscale FTIR spectra were collected by a neaSNOM instrument (Neaspec/Attocube, Germany) used in the scattering-type scanning near-field optical microscopy (s-SNOM) mode in the spectral regions of ca. 2000–1300 cm^−1^ (broadband IR laser, $\:\sim$1.06 mW) and ca. 1800–1000 cm^−1^ (broadband IR laser, $\:\sim$0.5 mW) at the second harmonic of the tip oscillation frequency. The instrument was aligned at the third harmonic to optimise near-field detection using the silicon surface of the AFM reference calibration grating “Test Grating TGQ1” reference (TipsNano, Estonia). The reference spectra were recorded on pure silicon. Pt/Ir coated monolithic ARROW-NCPt Si tips (NanoAndMore GmbH, Germany) with a tip radius < 10 nm (tapping frequency $\:\sim$275 kHz) were used for collecting the nanoscale FTIR spectra and provided a spatial resolution of <20 nm. Tapping amplitudes in contact were ca. 60 nm. Each spectrum was averaged from 10 interferograms with 512 mirror positions in each interferogram, the integration time at each mirror position was 9.8 ms the zero-filling factor used in the Fourier transformation was 4, and the spectral resolution was 16 cm^−1^. Recording of one spectrum took thus 50 s. Spectra were measured five times at the same spot. Phase tilt and phase offset corrections were applied to each spectrum, and then the average spectrum was calculated using the neaPLOT software (Neaspec, Germany). Spectra from two different spectral ranges were carefully combined also with the neaPLOT software and plotted in OMNIC™ 9 (ThermoFisher Scientific, USA).

#### EPS content assays

##### Degradation of bacterial Flocs

A bacterial culture with an O.D. of 0.5 was aliquoted into tubes, and each sample was incubated at 37 °C for 20 min with 59 µM of compound **8** to induce flocculation. Following incubation, the samples were centrifuged at 1500 rpm for 10 min (using a Thermo Scientific^®^ IEC CL31R Multispeed), and the resulting pellets were washed twice with saline (0.9% NaCl). Each pellet was then resuspended in 2 mL of a solution designed for specific degradation: cellulase (2 mg/mL in MES buffer, from *Trichoderma reesei*, Abnova) or periodic acid (0.4 M in Milli-Q water, VWR) for sugar degradation; proteinase K (2 mg/mL in Tris buffer, from *Tritirachium album*, Merck) for protein degradation; or DNase I (0.6 mg/mL in Tris-HCl buffer, bovine desoxyribonuclease I from pancreas, PanReac AppliChem) for DNA degradation. The samples were then incubated with agitation at 37 °C for 30 min. After incubation, the flocs visual integrity was assessed for qualitative analysis.

#### Interaction with bacterial membranes

##### Paramagnetic liposomal model

The liposomal suspension ([PO_4_^3−^] = 2.81 mM) was diluted 5-fold in distilled water, and samples were prepared to achieve a final compound **8** concentration of 59 µM, with a DMSO content of 5.9%. A 300 µL portion of this solution was placed in a thermostated glass tube and incubated for 30 min at 37 °C. Two control samples were also prepared and incubated under the same conditions: one with 5.9% DMSO as a negative control, and another with Triton X-100 (a 10-fold diluted commercial solution) as a positive control. Following incubation, the longitudinal relaxation time (T_1_) of each sample was measured at 37 °C using a Minispec mq 60 spin analyzer (Bruker^®^, Mannheim, Germany) operating at 60 MHz (1.41 T). Relaxivity *r*_1_ was calculated from the experimentally measured relaxation time T_1_ using the following equation (Eq. (2)):

2 $$\:\frac{1}{{\varvec{T}}_{1}}=\:\frac{1}{{\varvec{T}}_{1\left(\varvec{w}\varvec{a}\varvec{t}\varvec{e}\varvec{r}\right)\:}}+\:{\varvec{r}}_{1}\times\:\left[\varvec{G}\varvec{d}\right]$$

where $\:{\varvec{T}}_{1}$ is the relaxation time measured in the presence of the contrast agent, $\:{\varvec{T}}_{1\left(\varvec{w}\varvec{a}\varvec{t}\varvec{e}\varvec{r}\right)\:}$, is the relaxation time of pure water, $\:{\varvec{r}}_{1}$, is the longitudinal relaxivity (in s^−1^ mM^−1^), which represents the effectiveness of the contrast agent, and $\:\left[\varvec{G}\varvec{d}\right]$ is the Gd concentration (in mM).

##### Computational assays

All three studied systems were prepared using CHARMM-GUI and modelled with the all-atom CHARMM36m force field^49^, the CHARMM General Force Field (CGenFF)^50^ and the TIP3P water model^51^. Parameters for the bisbenzimidazole derivative (compound **8**) were generated using CGenFF v2.4.04^52^. The complexes were initially pre-equilibrated in the NVT ensemble before undergoing 1 microsecond of conventional molecular dynamics (MD) in the NPT ensemble using NAMD 2.14 engine^53^. Temperature and pressure were maintained at 300 K and 1 atm, respectively, employing overdamped Langevin dynamics and the Langevin piston^54,55^. A 4-fs time step was used to integrate the equations of motion, enabled by hydrogen mass repartitioning (HMR), which increases the mass hydrogen atoms by redistributing mass from their bonded heavy atoms while conserving the overall molecular mass^56–58^. Short-range interactions were smoothly tapered to zero between 10 and 12 Å, with a pair-list cutoff of 14 Å. Long-range electrostatic interactions were computed using the particle-mesh Ewald (PME) method^59^.

#### Bacterial mortality

##### Live/Dead BacLight^®^ bacterial viability

Bacterial cultures were grown to an optical density (O.D.) of 0.5, then incubated for 30 min at 37 °C either with 59 µM of compound **8** or with 5.9% DMSO (control). After incubation, aliquots were processed for both quantitative analysis and fluorescence microscopy. For quantitative viability assessment, samples were diluted 20-fold in phosphate-buffered saline (PBS, pH 7.4) and stained using the Live/Dead BacLight^®^ Bacterial Viability Kit (Molecular Probes, Invitrogen), following the manufacturer’s protocol optimised for fluorescence spectroscopy. Fluorescence measurements were recorded at 500 nm (SYTO 9, live cells) and 635 nm (propidium iodide, dead cells) using a Perkin Elmer LS-55 spectrofluorometer. A calibration curve was established using mixtures of live and isopropyl alcohol-killed bacteria to quantify the proportion of viable cells. For fluorescence microscopy, 1 mL of each bacterial suspension was centrifuged at 3000 rpm for 10 min and washed twice with sterile 0.9% NaCl. Cells were then stained by incubating the pellet with 3 µL/mL of the dye mixture (1:1 ratio of SYTO 9 and PI, as provided in the kit) for 15 min at room temperature in the dark. After staining, the samples were centrifuged and washed once more with saline. An aliquot of 20 µL of each bacterial suspension was deposited on a poly-L-lysine-coated microscope slide and allowed to air-dry under sterile conditions. Finally, 10 µL of saline was added before covering with a coverslip. Slides were imaged using a Leica^®^ DM2000 fluorescence microscope.

## Supplementary Information

Below is the link to the electronic supplementary material.

Supplementary Material 1

## Data Availability

All data generated or analysed during this study are included in this published article (and its Supplementary Information files).
