# Supplementary material for: Molecular self-assembly mediates the flocculation activity of benzimidazole derivatives against E. coli
Source: Sci Rep. 2025 Aug 5;15:28600. doi: 10.1038/s41598-025-13837-z (PMC12325786; doi:10.1038/s41598-025-13837-z)
Supplement: Supplementary file 1 — Supplementary Material 1 [file 41598_2025_13837_MOESM1_ESM.docx]

**Supporting information**

**Molecular Self-Assembly Mediates the Flocculation Activity of Benzimidazole Derivatives Against *E. coli***

**Isalyne Drewek ^1,2^, Aurélie Pietka ^1,2^, Thi Quynh Tran^3^, Marharyta Blazhynska^4^, Adéla Jeništova^5^, Christophe Chipot^4,6,7^, Andreas Barth^5^, Mathieu Surin^8^, Philippe Leclère^3^, Ruddy Wattiez^2^, Robert N. Muller^1,9^, Dimitri Stanicki^1*^, Sophie Laurent ^1,9^****^*^**

1. General, Organic and Biomedical Chemistry Unit (CGOB), Laboratory of Nuclear Magnetic Resonance and Molecular Imaging, Faculty of Medicine and Pharmacy, University of Mons - UMONS, B-7000 Mons, Belgium.
2. Laboratory of Proteomics and Microbiology, University of Mons - UMONS, B-7000 Mons, Belgium.
3. Laboratory for Physics of Nanomaterials and Energy, Research Institute for Materials, University of Mons, B-7000 Mons, Belgium.
4. Laboratoire International Associé Centre National de la Recherche Scientifique and University of Illinois at Urbana-Champaign, Unité Mixte de Recherche No 7019, Université de Lorraine, F-70239, Vandœuvre-lès-Nancy Cedex 54506, France.
5. Department of Biochemistry and Biophysics, The Arrhenius Laboratories for Natural Sciences, Stockholm University, S-106 91 Stockholm, Sweden.
6. Department of Biochemistry and Molecular Biology, The University of Chicago, Chicago, Illinois 60637, USA.
7. Theoretical and Computational Biophysics Group, Beckman Institute, and Department of Physics, University of Illinois at Urbana-Champaign, Urbana, Illinois 61801, USA.
8. Laboratory for Chemistry of Novel Materials, Centre of Innovation and Research in Materials and Polymers (CIRMAP), University of Mons - UMONS, B-7000 Mons, Belgium.
9. Center for Microscopy and Molecular Imaging (CMMI), B-6041 Charleroi, Belgium.

*Authors to whom correspondence should be addressed ([Dimitri.stanicki@umons.ac.be](mailto:Dimitri.stanicki@umons.ac.be) ; [sophie.laurent@umons.ac.be](mailto:sophie.laurent@umons.ac.be))

**Table of contents**

1. Supporting figures ……………………………………………………………………………………………………………. 2
2. Supporting tables ……………………………………………………………………………………………………………… 6
3. NMR spectra …………………………………………………………………………………………………………………….. 8
4. HRMS spectrum …………………………………………………………………………………………………………………. 9
5. Experimental protocols ……………………………………………………………………………………………………… 10
6. References ………………………………………………………………………………………………………………………… 14
7. **Supporting figures**

**

*Figure S1: Evolution of the bacterial population (%) of E. coli (K12-MG1655) in the presence of different DMSO percentages.*

*
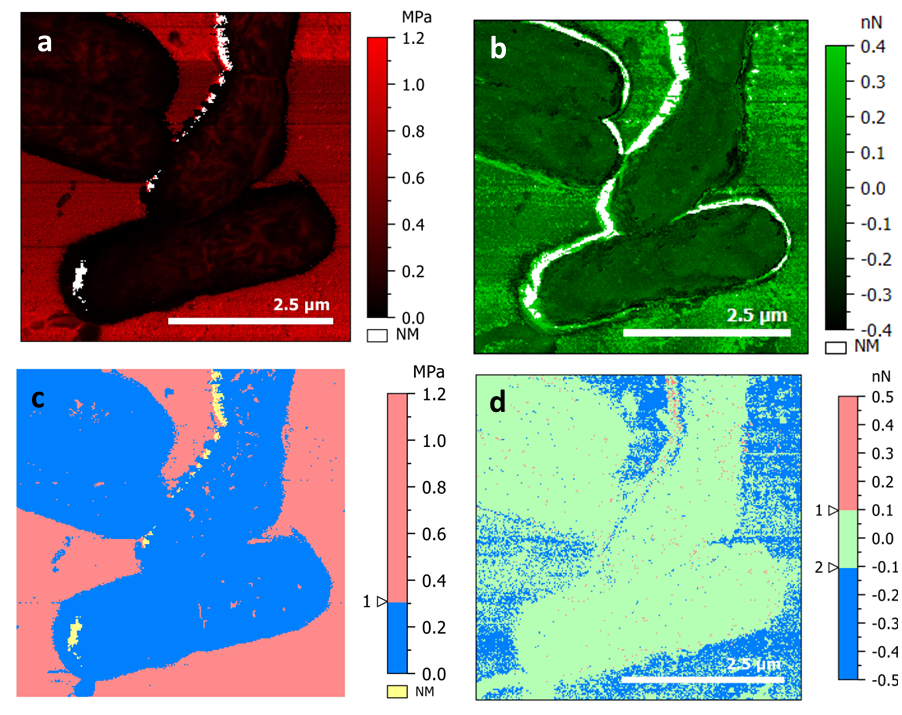
*

*Figure S2: Atomic force microscopy (AFM) studies using the Peak Force QNM (PFQNM) mode in fluid (deionised water). (a) Modulus image, (b) adhesion image, (c) cluster distribution of modulus image calculated based on image a, and (d) cluster distribution of adhesion image calculated based on image b. These measurements were performed on the control sample (E. coli cells). Areas marked “NM” (non-measured) correspond to pixels where mechanical properties could not be calculated due to insufficient or inconsistent force curve fitting. Scale bar in all images: 2.5 μm.*


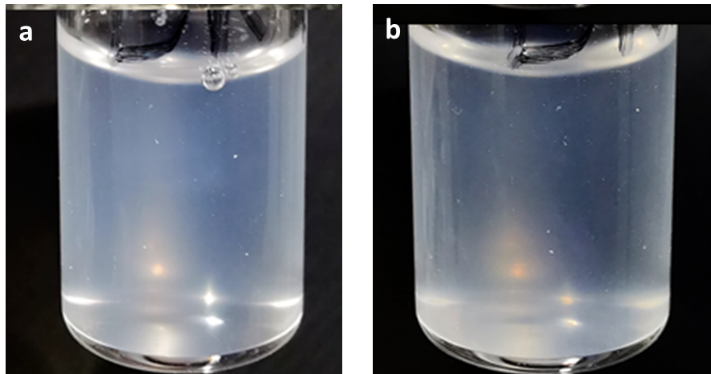


*Figure S3: Visual assessment of the impact of DNase I treatment on the integrity of E. coli flocs induced by compound* ***8****. Bacterial flocs were collected, resuspended, and incubated with DNase I (0.6 mg/mL) at 37 °C under gentle agitation. (a) Appearance of the floc suspension at the start of incubation (t = 0), (b) same sample after 30 minutes of incubation. No significant visual change was observed, indicating that extracellular DNA does not contribute to floc integrity.*

**a**

| Sample | Sugar content (μg/mL) |
| --- | --- |
| Control *E. coli* cells | 35.6 $\pm$ 1.2 |
| Flocculated *E. coli* cells | 29.6 $\pm$ 4.4 |

**b**

| Sample | Sugar content (μg/mL) |
| --- | --- |
| Control *E. coli* cells | 178.2 $\pm$ 59 |
| Flocculated *E. coli* cells | 191.4 $\pm$ 36.5 |

Figure S4: Calibration curved based on varying glucose concentrations (left), and sugar concentrations for the two sample types determined from the calibration curve (right). Results obtained for (a) strongly bond EPS, and (b) weakly bound EPS.


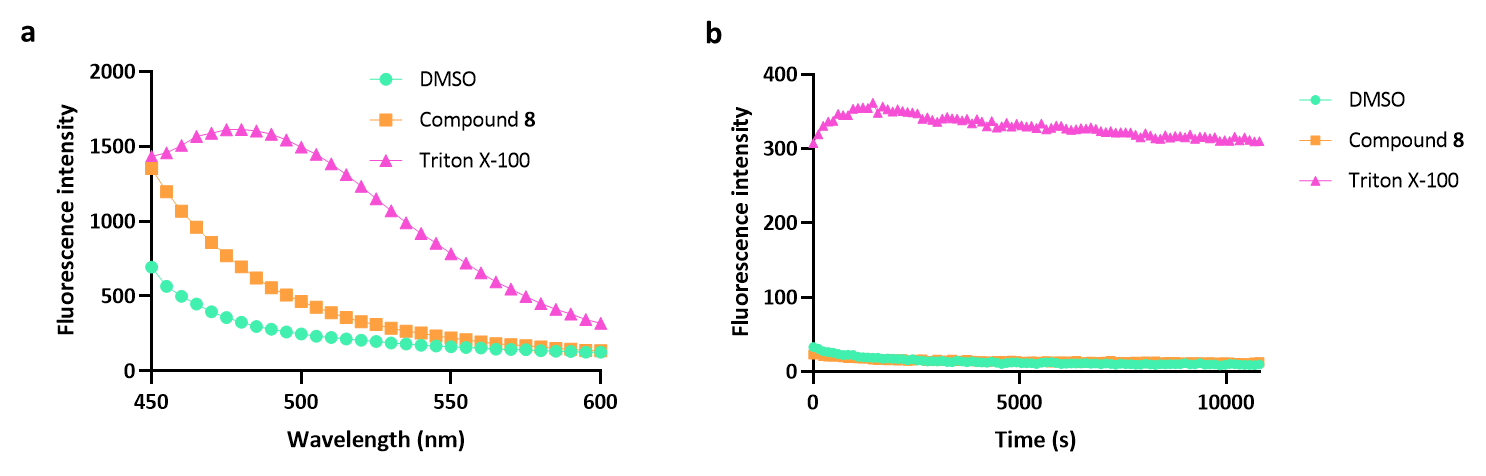


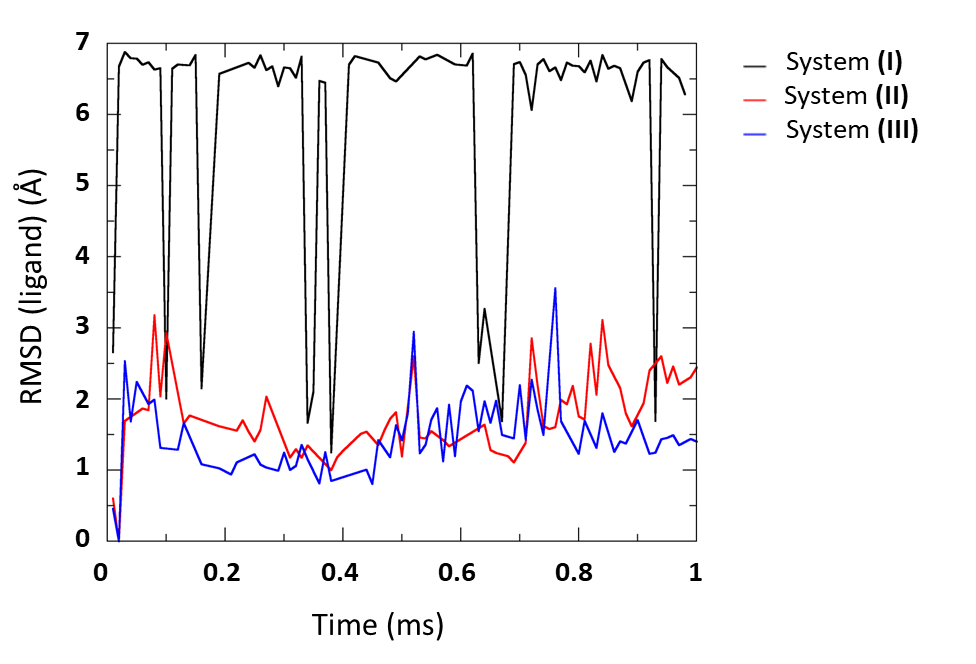
*Figure S5: Fluorescence spectra of E. coli treated with (a) ANS and 5.9% DMSO (negative control), Triton X-100 (positive control), or compound* ***8*** *(59 μM) for 30 minutes, and (b) DiSC_3_(5) and 5.9% DMSO (negative control), Triton X-100 (positive control), or compound* ***8*** *(59 μM) for 30 minutes. For both OM and IM, no integrity disruption was observed in the case of compound* ***8*** *and DMSO (negative control) while in the case of Triton X-100 (positive control), induced perturbation was confirmed.*

*Figure S6: Root Mean Square Deviation (RMSD) of compound* ***8*** *(ligand) excluding hydrogen atoms.*


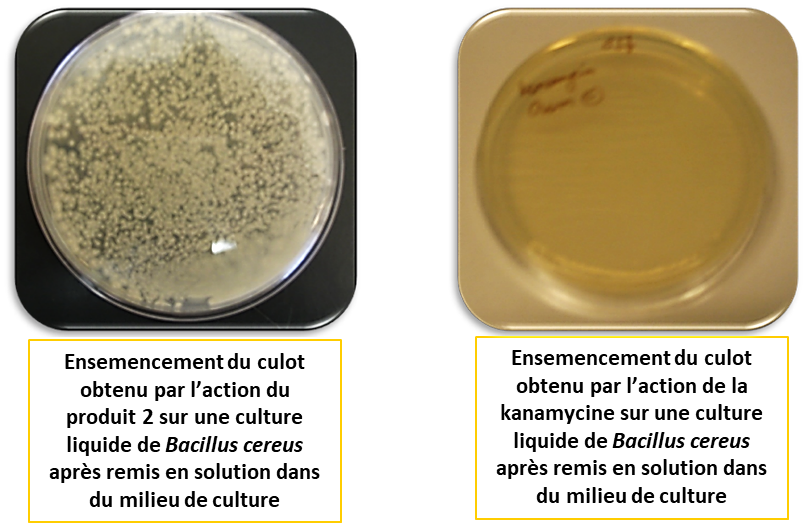


*Figure S7: Assessment of bacterial viability after flocculation induced by compound* ***8****. After flocculation of E. coli by compound* ***8****, the pellet (flocculated cells) was resuspended in LB medium, and plated on LB agar. Following overnight incubation at 37°C, numerous bacterial colonies were observed, indicating that the cells remained viable and capable of regrowth after treatment.*


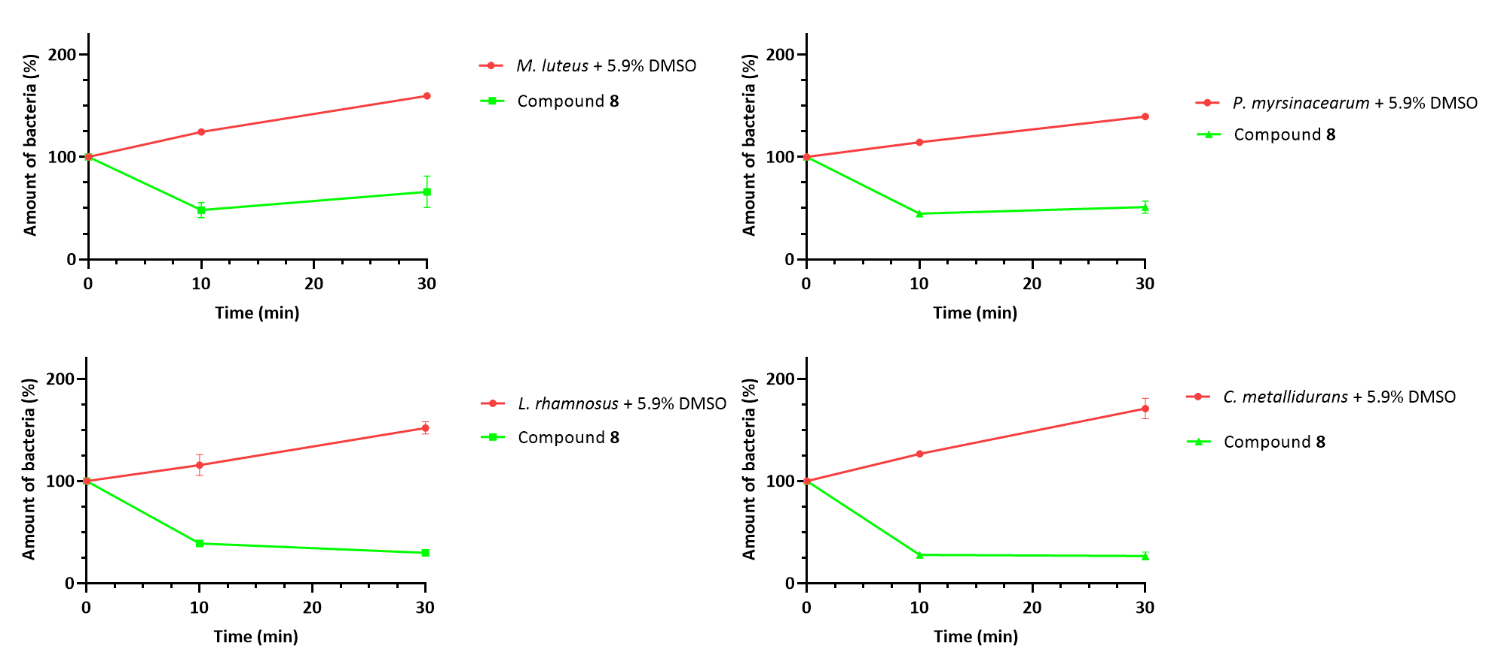


*Figure S8: Evolution of the bacterial population (%) of four bacterial strains (2 Gram-positive on the left and 2 Gram-negative on the right) in the presence of 5.9% of DMSO (control) and compound* ***8*** *(final standardised compound concentration of 59 µM).*

1. **Supporting tables**

*Table 1: Genes modified within the mutant strains and their specific features*

| Genes considered  (each gene corresponds to one bacterial strain used) | Specific features of these genes |
| --- | --- |
| ∆wcaA/∆wcaB/∆wcaC/∆wcaD | Genes that are part of a cluster of 20 genes encoding enzymes for colanic acid biosynthesis. Colanic acid is one of the extracellular polysaccharides secreted by bacteria into the surrounding environment^[1]^ |
| ∆pgaA/∆pgaB/∆pgaC/∆pgaD | The pga operon is accountable for the synthesis of poly-β-1,6-N-acetyl-D-glucosamine (or poly-β-1,6-GlcNAc, PGA). PGA is a polysaccharide that mediates cell-to-cell and cell-to-surface adhesion in biofilms. It is also essential for the formation of the non-random or periodic cellular architecture of *E. coli* biofilm microstructure^[2]^ |
| ∆csgA/∆csgB/∆csgC/∆csgD | The csg genes play a pivotal role in expressing proteinaceous fibril-like amyloid structures known as curli. Curli are crucial for cellular adhesion and facilitate host colonisation. These proteins foster intimate interactions with surfaces and assemble into inner-bacterial bundles, enabling a robust cell association within the biofilm^[3]^ |
| ∆yjbE/∆yjbF/∆yjbG/∆yjbH | The overexpression of the yjbEFGH operon leads to the secretion of exopolysaccharides in the case of *E. coli* K-12. These genes therefore contribute to the formation of biofilms. Nonetheless, their precise function remains incompletely understood^[4]^ |
| ∆rcs | The rcs system is one of the envelope stress response pathways in *E. coli*. These bacterial stress responses detect damage or defects in the cell envelope, which can result from environmental changes such as increased osmolarity, redox stress, or exposure to toxic molecules (e.g*.,* antibiotics), as well as intrinsic factors like errors in biosynthesis and protein misfolding. In response, these pathways alter the transcriptome to mitigate destructive stress. The rcs system specifically becomes activated by outer membrane (OM) damage, defects in lipopolysaccharides (LPS) synthesis, peptidoglycan disturbances, and mislocalisation of lipoproteins. This activation leads to changes in the expression of genes involved in capsule biosynthesis, motility, biofilm formation, and virulence^[5]^ |
| ∆cpx | The cpx system is another envelope stress response pathway found in *E. coli*. This system is triggered by defects in inner membrane (IM) protein secretion or by misfolding of IM or periplasmic proteins, which can occur due to various conditions, including changes in pH or osmolarity, cell adherence to hydrophobic surfaces, peptidoglycan biosynthesis defects, or exposure to copper^[6]^ |
| AgO^+^ | For this mutant, the presence of *O*-antigen on the surface of the *E. coli* outer membrane was restored |

Table S2: Bacterial strains used in the study, including name and genotype/collection.

| Strain name | Genotype / collection | Reference |
| --- | --- | --- |
| MG1655 | Wild-type reference strain | Lab collection |
| MG1655 ∆*cpx* | MG1655 ∆*cpxQPRA::FRT* | Rousseau et al., 2023, AAC^[7]^ |
| MG1655 ∆*rcs* | MG1655 ∆*rcsDB::FRT* | Rousseau et al., 2023, AAC^[7]^ |
| MG1665 AgO^+^ | MG1655 *wbbL^+^* | Lab collection |
| BW25113 | Wild-type, *lac*^-^ *ara*^-^ | Baba et al., 2006, MSB^[8]^ |
| BW25113 ∆*wcaA* | BW25113 ∆*wcaA::aphA2* | Baba et al., 2006, MSB^[8]^ |
| BW25113 ∆*wcaB* | BW25113 ∆*wcaB::aphA2* | Baba et al., 2006, MSB^[8]^ |
| BW25113 ∆*wcaC* | BW25113 ∆*wcaC::aphA2* | Baba et al., 2006, MSB^[8]^ |
| BW25113 ∆*wcaD* | BW25113 ∆*wcaD::aphA2* | Baba et al., 2006, MSB^[8]^ |
| BW25113 ∆*pgaA* | BW25113 ∆*pgaA::aphA2* | Baba et al., 2006, MSB^[8]^ |
| BW25113 ∆*pgaB* | BW25113 ∆*pgaB::aphA2* | Baba et al., 2006, MSB^[8]^ |
| BW25113 ∆*pgaC* | BW25113 ∆*pgaC::aphA2* | Baba et al., 2006, MSB^[8]^ |
| BW25113 ∆*pgaD* | BW25113 ∆*pgaD::aphA2* | Baba et al., 2006, MSB^[8]^ |
| BW25113 ∆*csgA* | BW25113 ∆*csgA::aphA2* | Baba et al., 2006, MSB^[8]^ |
| BW25113 ∆*csgB* | BW25113 ∆*csgB::aphA2* | Baba et al., 2006, MSB^[8]^ |
| BW25113 ∆*csgC* | BW25113 ∆*csgC::aphA2* | Baba et al., 2006, MSB^[8]^ |
| BW25113 ∆*csgD* | BW25113 ∆*csgD::aphA2* | Baba et al., 2006, MSB^[8]^ |
| BW25113 ∆yjbE | BW25113 ∆*yjbE::aphA2* | Baba et al., 2006, MSB^[8]^ |
| BW25113 ∆yjbF | BW25113 ∆*yjbF::aphA2* | Baba et al., 2006, MSB^[8]^ |
| BW25113 ∆yjbG | BW25113 ∆*yjbG::aphA2* | Baba et al., 2006, MSB^[8]^ |
| BW25113 ∆yjbH | BW25113 ∆*yjbH::aphA2* | Baba et al., 2006, MSB^[8]^ |
| *Lactobacillus rhamnosus* | ATCC 7469 | BCCM |
| *Cupriavidus metallidurans* | ATCC 43123 | BCCM |
| *Micrococcus luteus* | ATCC 4698 | BCCM |
| *Phyllobacterium myrsinacearum* | ATCC 43591 | BCCM |

1.
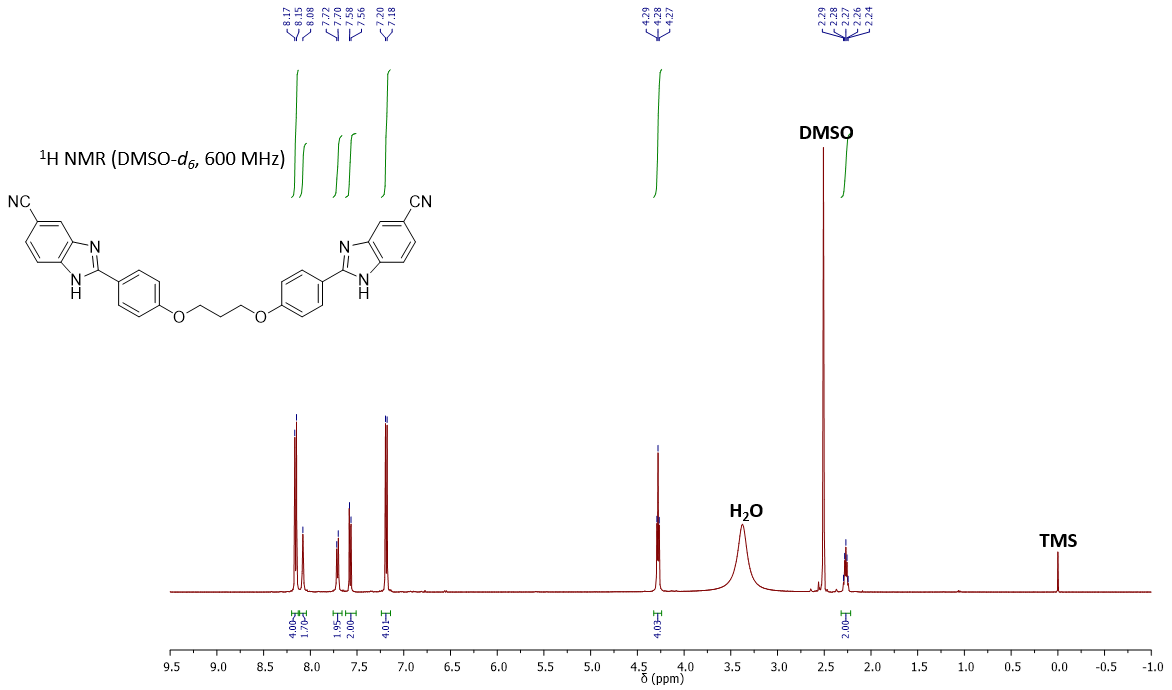
**NMR spectra**

**
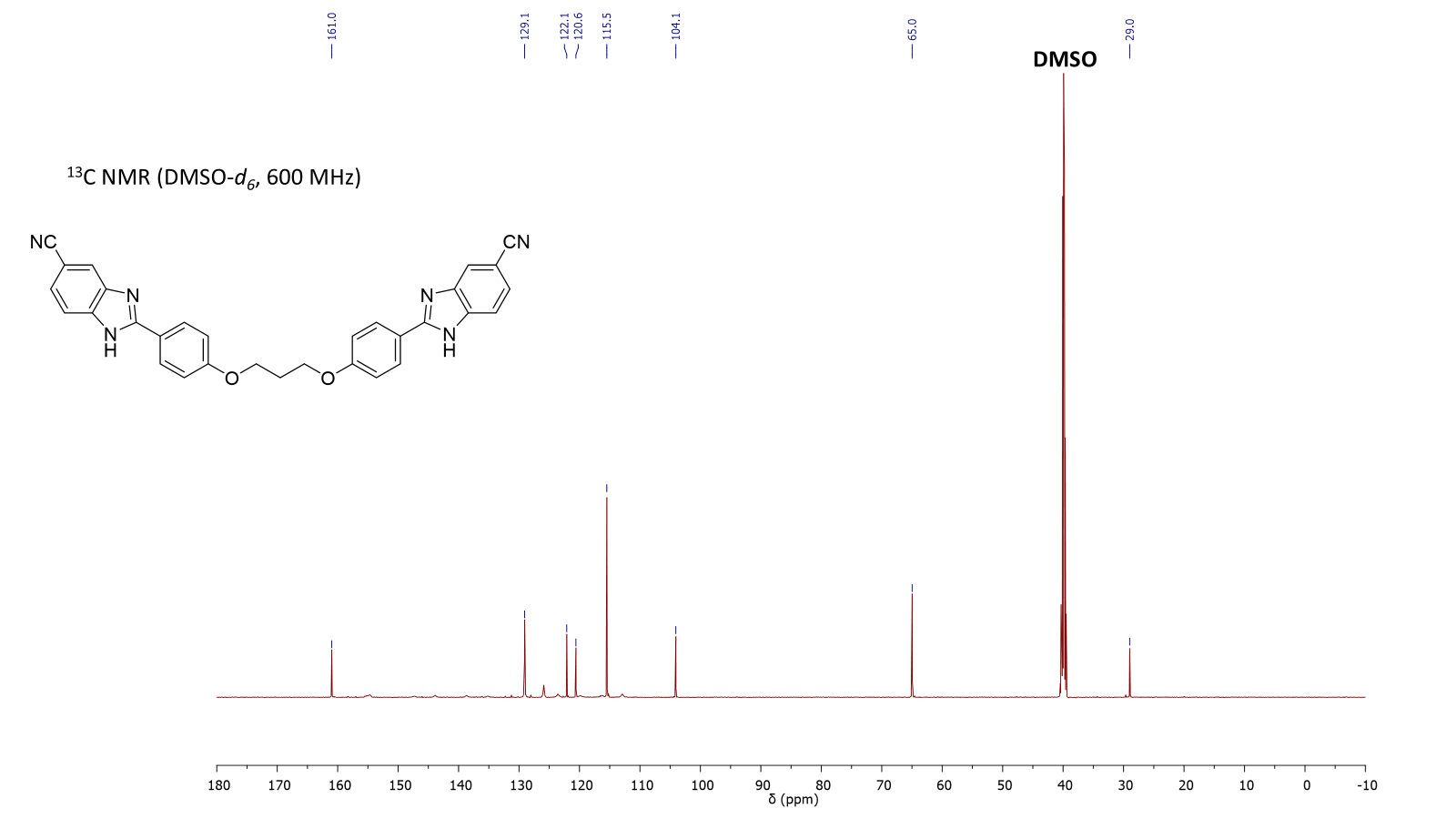
***Figure S9: ¹H NMR spectrum (600 MHz, DMSO‑d₆) of compound* ***8****: 2,2'-((propane-1,3-diylbis(oxy))bis(4,1-phenylene))bis(1H-benzo[d]imidazole-5-carbonitrile).*

*Figure S10: ¹^3^C NMR spectrum (600 MHz, DMSO‑d₆) of compound* ***8****: 2,2'-((propane-1,3-diylbis(oxy))bis(4,1-phenylene))bis(1H-benzo[d]imidazole-5-carbonitrile).*

1. **HRMS spectrum**

**
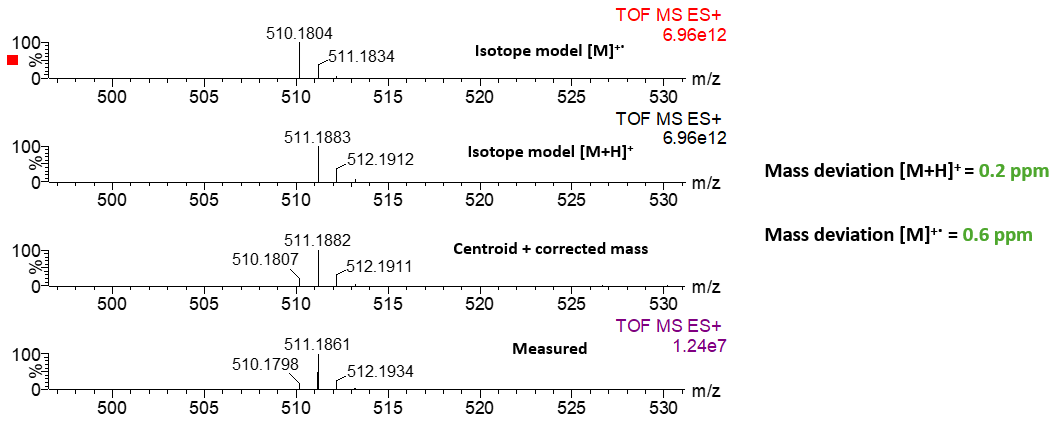
**

*Figure S11: High-resolution mass spectrum (HRMS, ESI⁺) of compound* ***8*** *showing the protonated molecular ion [M+H]⁺ and the radical cation [M]⁺^•^. For each ion species, the experimental pattern, the simulated isotopic distribution, and the centroid mass with corrected monoisotopic mass are shown. The measured masses are in agreement with the calculated values, confirming the molecular formula of compound* ***8****.*

1. **Experimental protocols**

**Confocal microscopy**

The bacterial strain used was *E. coli* K-12 MG1655, genetically modified to express Green Fluorescent Protein (GFP), resulting in a fluorescent cytosol. A bacterial culture with an O.D. of 0.5 was incubated for 15 minutes at 37°C with 59 μM of compound **8** or used as a control. After incubation, the samples were centrifuged for 10 minutes (3000 rpm for control and 1500 rpm for flocculated bacteria), and the pellets were washed with PBS. The samples were then fixed with paraformaldehyde for 10 minutes (4% solution in PBS) at room temperature, followed by a final PBS wash. The pellets were resuspended in 400 µL of PBS, and 100 µL of each sample was deposited on a poly-L-lysine-coated slide. After a 15-minute incubation, the slides were gently washed with PBS and allowed to dry completely. A drop of Dako Fluorescent Mounting Medium (Sigma Aldrich) was added to the dried sample, followed by a glass coverslip before microscopy.

All samples were imaged using a Zeiss® LSM710/AxioObserver Z1 confocal microscope with a Plan-Apochromat 63x/NA 1.4 oil DIC M27 immersion objective in a thermostatically controlled chamber (XL/LSM incubator, Zeiss; Tempcontrol 37-2, PeCon) set to 37°C. The images were acquired in z-stack mode (15 slices) with a slice thickness of 0.68 µm.

**Determination of sugar content using a colorimetric method**

Sugar extraction from bacterial flocs was carried out using a slightly modified protocol based on Eboigbodin *et al*^[9]^. A bacterial culture with an O.D. of 0.5 was treated with either 5.9% DMSO (control) or 59 μM of compound **8**. The control sample was placed on ice to stop bacterial growth, while the treated sample was incubated at 37°C with agitation for 15 min to induce flocculation. After incubation, all samples were centrifuged at 5000 rpm for 15 min at 4°C. The resulting pellet was used to extract strongly bound EPS, and the supernatant was used for weakly bound EPS extraction. For strongly bound EPS, the pellets were washed twice with saline (0.9% NaCl) and resuspended in a 1:1 solution of saline and 2% EDTA. The samples were incubated at 4°C for 1 h and then centrifuged at 10,000 g for 1 h at 4°C. The supernatants containing the strongly bound EPS were collected, filtered through a 0.45 µm cellulose membrane, and stored at 4°C for further analysis. For weakly bound EPS, the supernatants from the initial centrifugation were centrifuged at 10,000 g for 30 min at 4°C. The resulting supernatant was mixed with three volumes of absolute ethanol and stored at -20°C for 18 h to precipitate the EPS. After a 15-min centrifugation at 10,000 g and 4°C, the pellets were resuspended in Milli-Q water and dialysed for 2 days using 1 kDa cut-off membranes to remove ethanol. The dialysed samples were then freeze-dried, resuspended in Milli-Q water and dialysed for 2 days using 1 kDa cut-off membranes to remove ethanol. The dialysed samples were then freeze-dried, resuspended in Milli-Q water, and stored at 4°C for analysis.

For the colorimetric assay, the method of Dubois *et al.* was used^[10]^. To 400 µL of the EPS solution (either weakly or strongly bound) were added 10 µL of 80% phenol and 1 mL of concentrated sulfuric acid. The solutions were incubated at room temperature for 10 min with gentle stirring. The absorbance of the samples was measured at 490 nm using a SpectraMax® M2 Microplate Reader. A glucose calibration curve, ranging from 0 to 100 µg/mL, was also prepared for reference.

**Evaluation of the flocculation efficiency on mutants of the *E. coli* K-12 strain**

Nineteen mutant strains of *E. coli* K-12 BW25113/MG1655 (including ∆wcaA/∆wcaB/∆wcaC/∆wcaD; ∆pgaA/∆pgaB/∆pgaC/∆pgaD; ∆csgA/∆csgB/∆csgC/∆csgD; ∆yjbE/∆yjbF/∆yjbG/∆yjbH; ∆rcs; ∆cpx; AgO^+^) were sourced from the KEIO collection (NBRP-NIG, Japan). These strains were stored as aliquots at -80°C and re- cultured. The resulting 0.5 O.D. liquid cultures were distributed into 1 mL Eppendorf tubes and incubated with 59 μM of compound **8** at 37°C for 30 min. After incubation, the samples were visually inspected to qualitatively assess the flocculation response of each mutant strain.

**Preparation and characterisation of the liposomal suspension**

The preparation of liposomes followed the thin-film hydration technique, which, through several steps, yielded unilamellar vesicles of controlled size. A mixture of phospholipids (DPPC/DPPG, 7:3, 77 mg/33 mg) was combined with a chloroform/methanol mixture (7:3 v/v, 100 mL) in a flask. This solution was ultrasonicated to fully dissolve the phospholipids. Following solubilisation, the solvent was evaporated under reduced pressure until a thin lipid film formed on the flask wall. This lipid film was then rehydrated in an aqueous solution of ProHance® (a gadolinium complex, 6 mL volume, C used = 0.1 mmol/mL) while stirring mechanically at a temperature above the phase transition temperature (around 50°C) for over two hours, until the mixture turned milky. The resulting multilamellar liposomes were then extruded three times through polycarbonate filters with decreasing pore sizes (0.8, 0.4, and 0.2 µm) at a temperature above the phospholipid mixture phase transition temperature (60°C), producing a unilamellar liposomal suspension with a diameter between 180 and 200 nm. This suspension was dialysed against Milli-Q water for 3-4 days to remove unencapsulated ProHance® (12-14 kDa dialysis membrane) and then stored at 4°C. The liposomal suspension was characterised by Dynamic Light Scattering (DLS) using a Zetasizer Nano ZS particle size analyzer (He-Ne laser, 633 nm) from Malvern Instruments® (Worcestershire, UK) to determine the hydrodynamic diameter of the liposomes. The liposomal suspension was diluted 5-fold in distilled water, and the size distribution (% intensity) was measured. The results showed a low polydispersity index (PDI = 0.09) and an average hydrodynamic diameter of 180 nm, consistent with the extrusion processes employed during synthesis.

The phosphate concentration was determined using the method based on Bartlett’s assay^[11]^, as reviewed by Barenholz^[12]^. The liposomal suspension stock was first diluted 40-fold in distilled water to bring it within the concentration range specified by the protocol. A 100 µL aliquot of the diluted suspension was transferred to a Pyrex® tube and heated in a nitrite-nitrate bath (composed of 40% NaNO_2_, 7% NaNO_3_, and 53% KNO_3_) at 180°C until a dry residue formed. Subsequently, 0.3 mL of 70% perchloric acid was added, and heating continued at 180°C for an additional 45 minutes, with the tube sealed using a glass bead. After heating, the tube was allowed to cool before adding 1 mL of distilled water, followed by 0.4 mL of a 1.25% (w/v) hexa-ammonium molybdate solution, and 0.4 mL of a 5% (w/v) ascorbic acid solution. The mixture was vortexed and then heated in a boiling water bath for 5 minutes. After cooling, the absorbance of the solution was measured at 815 nm using a SpectraMax® M2 Microplate Reader. The phosphate concentration was determined using a calibration curve generated from a 0.5 mM NaH_2_PO_4_.H_2_O solution, covering a concentration range of 0 to 80 nmol phosphate. Using this method, a phosphate concentration of 14.05 ± 0.27 mM was determined for the liposomal suspension.

To determine the concentration of encapsulated Gd complexes, a digestion method was employed. The liposomal solution was initially diluted 25-fold in an aqueous solution containing 12% concentrated nitric acid (60%) and 6% hydrogen peroxide (35%). The mixture was then subjected to microwave digestion, after which it was cooled and collected. The resulting solution was further diluted 2-fold with distilled water (leading to a 6% acid matrix), and the Gd concentration was measured using ICP-AES (Varian Liberty Series II instrument, Varian Inc.®, Palo Alto, USA). Using this method, a Gd concentration of 7.14 ± 0.09 mM was determined.

**Membrane integrity assessment using fluorescent studies**

*Outer membrane permeability assay*

A bacterial culture with an initial O.D. of 0.5 was pelleted, washed several times with a Tris buffer (10 mM Tris HCl, 150 mM NaCl, pH 7.4), and resuspended in the buffer to achieve a final O.D. of 0.05. The culture was then pelleted again and resuspended in Tris buffer containing 10 µM ANS (8-anilinonaphthalene-1-sulfonic acid). The ANS-treated culture was dispensed into a sterile 96-well plate (Thermo Scientific®), and compound **8** was added to reach a final concentration of 59 μM in three distinct wells. Two control samples were also prepared: one with 5.9% DMSO as a negative control and another with 6% Triton X-100 as a positive control. The plate was shaken at room temperature for 20 minutes to equilibrate the cells, after which fluorescence intensity was recorded between 450 and 600 nm at an excitation wavelength of 380 nm at different incubation times (0, 30, 60, and 90 minutes at 37°C) using a SpectraMax® M2 Microplate Reader.

*Inner membrane depolarisation assay*

A bacterial culture with an initial O.D. of 0.5 was pelleted, washed several times with an HEPES buffer (5 mM sodium HEPES, 2 mM EDTA, pH 8), and resuspended in the buffer to achieve a final O.D. of 0.05. The culture was then pelleted again and resuspended in HEPES buffer containing 1 µM DiSC_3_(5) (3,3’-dipropylthiadicarbocyanine iodide). The DiSC_3_(5)-treated culture was dispensed into a sterile 96-well plate (Thermo Scientific®), and compound **8** was added to reach a final concentration of 59 μM in three distinct wells. Two control samples were also prepared: one with 5.9% DMSO as a negative control and another with 6% Triton X-100 as a positive control. The plate was shaken at room temperature for 60 minutes, followed by a 20-minute incubation at 37°C to induce flocculation. Fluorescence intensity at an emission wavelength of 670 nm was then measured over time with an excitation wavelength of 622 nm, all at room temperature using a SpectraMax® M2 Microplate Reader.

1. **References**

[1] Wang, C. *et al.* Colanic acid biosynthesis in Escherichia coli is dependent on lipopolysaccharide structure and glucose availability. *Microbiol. Res.* **239**, 126527 (2020).

[2] Itoh, Y. *et al.* Roles of *pgaABCD* Genes in Synthesis, Modification, and Export of the *Escherichia coli* Biofilm Adhesin Poly-β-1,6- *N* -Acetyl- d -Glucosamine. *J. Bacteriol.* **190**, 3670–3680 (2008).

[3] Khambhati, K., Patel, J., Saxena, V., A, P. & Jain, N. Gene Regulation of Biofilm-Associated Functional Amyloids. *Pathogens* **10**, 490 (2021).

[4] Ferrières, Lionel., Aslam, S. N., Cooper, R. M. & Clarke, D. J. The yjbEFGH locus in Escherichia coli K-12 is an operon encoding proteins involved in exopolysaccharide production. *Microbiology* **153**, 1070–1080 (2007).

[5] Meng, J., Young, G. & Chen, J. The Rcs System in Enterobacteriaceae: Envelope Stress Responses and Virulence Regulation. *Front. Microbiol.* **12**, 627104 (2021).

[6] Raivio, T. L. Everything old is new again: An update on current research on the Cpx envelope stress response. *Biochim. Biophys. Acta BBA - Mol. Cell Res.* **1843**, 1529–1541 (2014).

[7] Rousseau, C. J., Fraikin, N., Zedek, S. & Van Melderen, L. Are envelope stress responses essential for persistence to β-lactams in *Escherichia coli* ? *Antimicrob. Agents Chemother.* **67**, e00329-23 (2023).

[8] Baba, T. *et al.* Construction of *Escherichia coli* K‐12 in‐frame, single‐gene knockout mutants: the Keio collection. *Mol. Syst. Biol.* **2**, 2006.0008 (2006).

[9] Eboigbodin, K. E. & Biggs, C. A. Characterization of the Extracellular Polymeric Substances Produced by *Escherichia coli* Using Infrared Spectroscopic, Proteomic, and Aggregation Studies. *Biomacromolecules* **9**, 686–695 (2008).

[10] DuBois, Michel., Gilles, K. A., Hamilton, J. K., Rebers, P. A. & Smith, Fred. Colorimetric Method for Determination of Sugars and Related Substances. *Anal. Chem.* **28**, 350–356 (1956).

[11] Bartlett, G. R. Phosphorus Assay in Column Chromatography.

[12] Barenholz, Y. & Amselem, S. Quality control assays in the development and clinical use of liposome-based formulation. in *Liposome Technology, 2nd edition, Liposome Preparation and Related Techniques* vol. 1 (CRC Press, 1993).
